# Matrix stiffness boosts PDAC chemoresistance via SCD1-dependent lipid metabolic reprogramming

**DOI:** 10.1093/rb/rbaf056

**Published:** 2025-06-16

**Authors:** Xue Zhang, Biwen Zhu, Jiashuai Yan, Xi Chen, Di Wu, Zhen Wang, Xiaoqi Guan, Yan Huang, Yahong Zhao, Yumin Yang, Yibing Guo

**Affiliations:** Research Center of Clinical Medicine, Affiliated Hospital of Nantong University, Nantong University, Nantong 226001, PR China; Key Laboratory of Neuro-regeneration of Jiangsu and Ministry of Education, Co-innovation Center of Neuro-regeneration, Nantong University, Nantong 226001, PR China; Research Center of Clinical Medicine, Affiliated Hospital of Nantong University, Nantong University, Nantong 226001, PR China; Department of Hepatobiliary and Pancreatic Surgery, Affiliated Hospital of Nantong University, Medical School of Nantong University, Nantong 226001, PR China; Research Center of Clinical Medicine, Affiliated Hospital of Nantong University, Nantong University, Nantong 226001, PR China; Department of Hepatobiliary and Pancreatic Surgery, Affiliated Hospital of Nantong University, Medical School of Nantong University, Nantong 226001, PR China; Research Center of Clinical Medicine, Affiliated Hospital of Nantong University, Nantong University, Nantong 226001, PR China; Key Laboratory of Neuro-regeneration of Jiangsu and Ministry of Education, Co-innovation Center of Neuro-regeneration, Nantong University, Nantong 226001, PR China; Research Center of Clinical Medicine, Affiliated Hospital of Nantong University, Nantong University, Nantong 226001, PR China; Department of Hepatobiliary and Pancreatic Surgery, Affiliated Hospital of Nantong University, Medical School of Nantong University, Nantong 226001, PR China; Research Center of Clinical Medicine, Affiliated Hospital of Nantong University, Nantong University, Nantong 226001, PR China; Department of Hepatobiliary and Pancreatic Surgery, Affiliated Hospital of Nantong University, Medical School of Nantong University, Nantong 226001, PR China; Research Center of Clinical Medicine, Affiliated Hospital of Nantong University, Nantong University, Nantong 226001, PR China; Department of Hepatobiliary and Pancreatic Surgery, Affiliated Hospital of Nantong University, Medical School of Nantong University, Nantong 226001, PR China; Research Center of Clinical Medicine, Affiliated Hospital of Nantong University, Nantong University, Nantong 226001, PR China; Department of Hepatobiliary and Pancreatic Surgery, Affiliated Hospital of Nantong University, Medical School of Nantong University, Nantong 226001, PR China; Key Laboratory of Neuro-regeneration of Jiangsu and Ministry of Education, Co-innovation Center of Neuro-regeneration, Nantong University, Nantong 226001, PR China; Key Laboratory of Neuro-regeneration of Jiangsu and Ministry of Education, Co-innovation Center of Neuro-regeneration, Nantong University, Nantong 226001, PR China; Research Center of Clinical Medicine, Affiliated Hospital of Nantong University, Nantong University, Nantong 226001, PR China

**Keywords:** matrix stiffness, lipid metabolism, chemoresistance, stearoyl-CoA desaturase 1, pancreatic ductal adenocarcinoma

## Abstract

PDAC cells perceive and respond to mechanical stimuli in their extracellular microenvironments (ECMs), playing a crucial role in chemoresistance, while the underlying mechanisms are not fully understood. The progression of various solid tumors is accompanied by metabolic reprogramming. RNA-seq and untargeted metabolomics analysis indicated that stiff substrate may regulate lipid metabolism. The expression of lipogenesis-related genes, including fatty acid synthase (FASN), ATP citrate lyase (ACLY) and acetyl-CoA carboxylase (ACC) was elevated, also the sum of lipid droplets and the triglyceride content. Herein, whether lipid metabolism is involved in matrix stiffness-mediated PDAC chemoresistance and the in-depth mechanism were further explored. Rescue with C75 (FASN inhibitor) validated that fatty acid synthesis participated in matrix stiffness-regulated chemoresistance. Simultaneously, the SCD1 expression was reinforced, consistent with PDAC tissues. The concurrent restraint SCD1 (with inhibitor CAY10566 or shSCD1) and addition of oleic acid confirmed that SCD1 is involved in matrix stiffness-mediated chemoresistance through fatty acid synthesis. In addition, Piezo1 regulated SCD1 expression through the augmentation of Ca^2+^ influx, and the PI3K/Akt pathway participated in this process. Taken together, our research sheds light on lipid metabolism exerts an essential role during matrix stiffness-mediated chemoresistance through Piezo1-elicited elevation of SCD1. Our findings delivered a supplement PDAC chemoresistance mechanism mediated by matrix stiffness from the perspective of lipid metabolic reprogramming, and provided a novel strategy for improving clinical therapies.

## Graphical abstract

**Figure:**
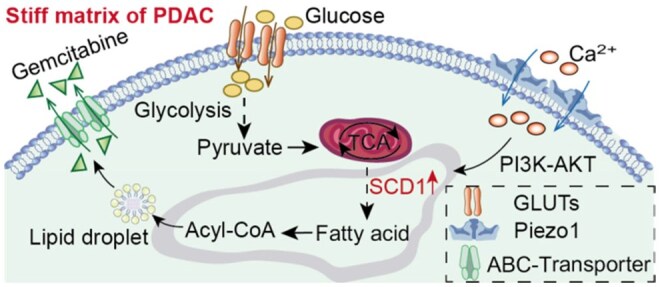

## Introduction

Pancreatic ductal adenocarcinoma (PDAC) is featured by stiffening desmoplasia, with lower 5-year survival rates and higher mortalities, which is partially associated with acquired chemoresistance. Recent advancements in available mechanical-tunable materials such as polyacrylamide, gelatin methacryloyl (GelMA), polyethylene glycol and hyaluronic acid methacrylate have been generally utilized to simulate the matrix stiffness of various solid tumors, which further impelled the initiation and progression via regulating assorted cellular behavior. For PDAC, higher matrix stiffness promoted chemoresistance based on an adjustable three-dimensional (3D) hydrogel composed of hyaluronic acid and elastin-like protein [1]. In addition, autophagy involved in reinforcing PDAC chemoresistance within the GelMA hydrogel model matched the pathological matrix stiffness [2]. While for breast cancer, stiffer fibronectin-coated substrates impair the efficacy of etoposide and cisplatin through the repair of dsDNA breaks [3].

The hallmark of metabolic reprogramming exerted a vital function during PDAC occurrence and progression. Specifically, lipid metabolic reprogramming has initially attracted attention in mediating tumor chemoresistance [4]. Cancer cells enhanced survival, proliferation and resistance to drug treatment through alteration of lipid metabolism, which includes lipid synthesis, storage and degradation [5]. For instance, enhanced fatty acid (FA) synthesis promoted cholangiocarcinoma progression and chemoresistance via elevating poly ADP-ribose polymerase-dependent HIF-1α deubiquitylation [6]. Additionally, pre-treatment with exogenous FAs increased the gemcitabine resistance in PDAC [7]. Current research has manifested that the biophysical parameter of the tumor niche also mediates the alteration of lipid metabolism. In hepatocellular carcinoma (HCC), extracellular matrix stiffness-triggered lipid metabolic reprogramming through mechano-reactive long-chain acyl-CoA dehydrogenase, which initiated FA oxidation [8]. Furthermore, the soft matrix primed triple negative breast cancer cells to store significantly lipid droplets (LDs) and heighten lipid metabolism via the citric acid cycle [9]. However, the effect of lipid metabolic reprogramming in matrix stiffness-mediated PDAC chemoresistance needs to be further elucidated.

FAs comprise monounsaturated (MUFAs) and saturated forms, which play vital roles in cellular heterogeneity and neoplasm growth [10]. As an anabolic process, FAs synthesis depended on the tandem-activation of the acetyl-CoA carboxylase (ACC), ATP citrate lyase (ACLY) and fatty acid synthase (FASN) [5]. As an essential mechano-mediator and rate-limiting enzyme, stearoyl-CoA desaturase 1 (SCD1) participated in the production of MUFAs, for instance oleic acid (OA) and palmitoleic acid [11]. SCD1 reprogrammed lipid metabolism to regulate the invasion of HCC cells [12]. In gastric cancer, SCD1 favored the formation of LDs to mitigate chemotherapy-induced endoplasmic reticulum stress and enhance self-renewal [13]. It is well known that several mechanosensitive elements exerted momentous regulators in PDAC pathological behavior and metabolic activity, such as yes-associated protein (YAP) and integrins. Piezo1, as a new mechanosensitive ion channel protein, regulates various biological processes by converting physical stimuli into electrochemical signals by accelerating Ca^2+^ influx. Emerging as a significant driver of cellular metabolism, Piezo1 may be involved in the PDAC metabolism mediated via mechanical stimuli.

Taken together, we proposed that lipid metabolism participated in matrix stiffness-mediated PDAC chemoresistance, and the underlying mechanism may be related to Piezo1-triggered SCD1. To confirm this hypothesis, a three-dimensional GelMA hydrogel model that matches the stiffness of healthy pancreas and PDAC tissues was established. Additionally, according to RNA-seq and untargeted metabolomics analysis, matrix stiffness-mediated lipid metabolism and the relationship with chemoresistance were also conducted. Finally, Piezo1-mediated SCD1 expression and the underlying pathway of PI3K-Akt were further carried out. Our research delineated lipid metabolic reprogramming adaptation to PDAC chemoresistance from the perspective of the biomechanics, which provides an innovative therapeutic strategy via dismantling the metabolic cross-talk.

## Materials and methods

### Cell cultivation

Human-derived PDAC cell lines, CFPAC-1 and Mia-PaCa2, were procured from the Cell Bank of the Chinese Academy of Sciences. CFPAC-1 cells were incubated in Dulbecco's Modified Eagle Medium (DMEM) medium added with 10% FBS (35050061; Invitrogen, USA) and 1% penicillin/streptomycin (11360070; Invitrogen). While for Mia-PaCa2 cells, additionally supplemented with 3% horse serum (26050070; Gibco) and 1% sodium pyruvate (11360070; Gibco). Then cultured in the incubator (37°C, 5% CO_2_).

### Cells encapsulation within GelMA hydrogel

The precursor solution of GelMA (EFL-GM-60; Engineering For Life, Suzhou, China) was prepared as per the instructions. Specifically, the designated weight of GelMA was dissolved in the photoinitiator of 0.25% (w/v) LAP, and crosslinked with the final concentration of 5 × 10^6^ cells/mL. 5% (Soft group) and 10% (Stiff group) GelMA (w/v) with uniformly encapsulated cells were cured by 405 nm ultraviolet light for 10 and 30 s, respectively, which matched the stiffness scope of physiology (healthy) and pathology (tumor tissue) of PDAC.

### Nile red staining

Fixed with 4% paraformaldehyde (PFA), then stained with Nile red (30 μM; MCE, USA) for 15 min at 37°C and counterstained with DAPI (BL105A; Biosharp, China) to visualize the LDs with a confocal microscope (LSM900; Zeiss, Germany).

### Triglyceride assay

The level of triglycerides (TGs) was detected by Amplex Red Triglyceride Assay Kit (S0219S; Beyotime, China) as per the instructions. In short, the samples were diluted, then lipase was added dropwise and incubated for 20 min at 37°C. Following incubation with the TG detection working solution in the dark, the absorbance was measured at 570 nm.

### Quantitative Reverse Transcription Polymerase Chain Reaction (qRT-PCR)

Total RNA of PDAC cells was extracted with TRIzol reagent (343903; Invitrogen) as per the manufacturer’s protocol. Then transcribed through a cDNA reverse transcription kit (K1622; Thermo Fisher Scientific, USA) and amplified via the Fast SYBR Green qPCR Kit (1176202 K; Thermo Fisher Scientific), and relative gene expression was normalized to β-actin and calculated with the 2^−ΔΔCt^ method. Supplementary Table S1 lists the detailed primer sequences.

### Oil red O staining

The tissues were immobilized in 4% PFA, then embedded with OCT and sliced after pre-cooling the slicer. Treated with Oil Red staining solution (HY-D1168; MCE) for 10 min, then washed away excess staining solution with 70% ethanol. Tissues were counterstained with hematoxylin solution, and images were captured using a confocal microscope (LSM900).

### Transmission electron microscopy (TEM)

The harvested cells were fixed in 4% glutaraldehyde (111-30-8; Sigma, USA) for 2 h and resuspended with 1% agarose after discarding the supernatant. Then, the samples were rinsed with phosphate buffer and dehydrated with a gradient ethanol series. Following treatment with propylene oxide, they were immersed overnight with the embedding agent and sliced (70–90 nm thickness). The morphology of LDs was observed after staining with 4% uranium acetate and 0.3% lead citrate using TEM (HT7700; Hitachi).

### Immunofluorescence assays

Cells were fixed with 4% PFA and then permeabilized with 0.2% Triton X-100 (T8200; Solarbio), further blocked with 10% Bovine Serum Albumin (BSA) at 37°C, and incubated with primary antibody overnight at 4°C. As follows: anti-SCD (1:200, 28678-1-AP; Proteintech), anti-ACLY (1:100, 67166-1-lg; proteintech), anti-FASN (1:200, 10624-2-AP; Proteintech), anti-ACC1 (1:100, 21923-1-AP; Proteintech), anti-ABCC1 (1:100, ab233383; Abcam), anti-ABCC3 (1:200, ab204322; Abcam) and anti-Piezo1 (1:200, 15939-1-AP; Proteintech). The following day, washed with Phosphate-Buffered Saline with Tween 20 (PBST), then incubated with secondary antibody: Goat anti-rabbit IgG H&L Alexa Fluor 647 (1:1000, ab150079; Abcam) or Goat anti-Mouse IgG H&L Alexa Fluor 594 (1:1000, ab150077; Abcam). The immunofluorescence images were captured on a confocal microscope (LSM900). The quantitative analysis was carried out with ImageJ.

### Immunohistochemistry staining

The adjacent and PDAC tissues with patient informed consent were acquired from the Affiliated Hospital of Nantong University, and the experimental protocol was approved and granted by the ethics committee (No. 2021-K136). Hydrogels encapsulated with Mia-PaCa2 cells from nude mice and patient tissues were both fixed in 10% neutral-buffered formalin. The specimens were paraffin-embedded and sliced (4 μm, thickness). Sections were deparaffinized and rehydrated, then treated with 3% H_2_O_2_ and blocked. Following incubation with the primary antibodies, the secondary antibodies and diaminobenzidine (DAB) staining in turn. The images were scanned on a confocal microscope (LSM900). The primary and secondary antibodies: anti-FASN (1:1000), anti-ACC1 (1:100), anti-ACLY (1:1000) and anti-SCD (1:200). Goat Anti-Rabbit IgG H&L (Horseradish Peroxidase, HRP) (1:10 000, ab205718; Abcam) and Goat Anti-Mouse IgG H&L (HRP) (1:10 000, ab205719; Abcam).

### Western blotting

The harvested cells were extracted with RIPA lysis buffer (EpiZyme, PC101, China) with cocktail inhibitor (Beyotime) to restrain protease and phosphatase. The proteins were separated and transferred to PVDF membranes (Merck Millipore, USA). Following blocking with 5% skimmed milk, the samples were incubated with primary and secondary antibodies at 4°C and room temperature, respectively. Based on markers (KGB3109-250; KeyGEN BioTECH, China), protein bands were visualized with Chemiluminescence (Biosharp, China). Quantitative analysis of gray value was conducted with ImageJ. The primary antibodies: anti-ABCC1 (1: 1000), anti-ABCC3 (1:2000), anti-FASN (1:5000), anti-ACC1 (1:5000), anti-ACLY (1:5000), anti-SCD (1:5000), anti-Piezo1 (1:500), anti-PI3K (1:1500, CY6915; Abways), anti-p-PI3K (1:1500, CY6427; Abways), anti-Akt (1:5000, 10176-2-AP; Proteintech) and anti-p-Akt (1:5000, 66444-1-Ig; Proteintech). The secondary antibodies:HRP-Goat Anti-Rabbit Recombinant Secondary Antibody (H + L) (ZYID002-0050; Zunyan, Nanjing, China) and HRP-Goat Anti-Mouse Recombinant Secondary Antibody (H + L) (ZYID001-0050). GAPDH was used as the internal reference protein.

### Cell viability assay

Cells were treated with gemcitabine (range 0.01–1000 μM) for 48 h, and cytotoxicity was assessed. Each well was added with Cell Counting Kit-8 (CCK-8) (C6005; NCM Biotech) and incubated. The Optical Density (OD) values at 450 nm were measured, and the IC_50_ value was calculated by logarithmic regression using GraphPad Prism 9 software.

### RNA sequencing

The Mia-PaCa2 cells encapsulated in 5% and 10% GelMA hydrogels were lysed with TRIzol reagent (343903; Invitrogen), and a NanoDrop 2000 spectrophotometer was used to evaluate the RNA purity and integrity. Each group was sequenced with three replicates. The cDNA library was constructed via PCR amplification of the fragments, and the resulting products were purified. The sequencing process was conducted with the MGI high-throughput sequencer. The RNA sequencing data were then evaluated by the BGISEQ platform (Helix Life, Shanghai, China). The analyses of GO and KEGG with differential expression genes (DEGs, *P*_adj_ < 0.05, |Log_2_FC| > 1.5) were implemented with DAVID (https://david.ncifcrf.gov). Gene set enrichment analysis (GSEA) was further applied to functional analysis of the full gene list. Data visualization with bioinformatics.com.cn.

### Intervention assay

For the stiff hydrogel group, PDAC cells were treated with 5 μM C75 (HY-12364; MCE) to inhibit the level of FA synthesis, handled with CAY10566 (SCD1 inhibitor, HY-15823; MCE) to inhibit the expression of SCD1, and 10 μM OA (HY-N1446; MCE) to increase FAs. For the rescue assay of Piezo1, the soft group was treated with 10 μM Yoda1 (Piezo1 promoter, HY-18723; MCE) and the stiff group was tackled with 5 μM GsMTx4 (Piezo1 inhibitor, HY-P1410; MCE). 10 μM SC79 (Akt promoter, HY-18749; MCE) and 5 μM GsMTx4 were added in the stiff group to evaluate SCD1 expression.

### Cell transfection

Plasmid transfection was carried out with Lipofectamine™ 3000 as per the protocol. After 24 h of infection, replace with a fresh culture medium. Transfection efficiency was assessed after 48 h. SCD1 shRNA was obtained from Genepharma (Suzhou, China), with the following sequences: Negative control: 5′-GTTCTCCGAACGTGTCACGTCAAGAGATTACGTGACACGTTCGGAGAATT-3′. shSCD1#1: 5′-GCTAGACTTGTCTGACCTAGATTCAAGAGATCTAGGTCAGACAAGTCTAGCTT-3′; shSCD1#2: 5′-GACCCCACCTACAAGGATAAGTTCAAGAGACTTATCCTTGTAGGTGGGGTCTT-3′; shSCD1#3: 5′-GTTCCAGAGGAGGTACTACAATTCAAGAGATTGTAGTACCTCCTCTGGAACTT-3′.

### Metabolomics and data analysis

The harvested cells were added with pre-cooled metabolite extraction solution ACN/MeOH/H_2_O (2:2:1). Liquid chromatography using Waters ACQUITY^®^ UPLC HSS T3 column for separation. The processing of mass spectrometry data is carried out using Compound Discoverer 3.1 software, involving peak extraction, alignment, calibration and standardization. The accurate mass (mass tolerance <10 ppm) and MS/MS data (mass tolerance <0.02 Da) were utilized to identify the metabolites, then matched with HMDB, METLIN and other public databases and a self-built standard library (Bioprofile, China). R software (version 4.0.3) was applied to analyze multivariate data, and the VI*P* values were calculated based on OPLS-DA. Significant metabolites were screened by combining *P *< 0.05 and VIP > 1, and analyzed through volcano plot and heatmap analysis. The pathway analysis of metabolism was mapped to the KEGG with the R package (KEGGREST).

### *In vivo* experiment

The procedures were conducted with the guidelines of laboratory animals and were approved by the Animal Ethics Committee of Nantong University (No. S20210219-007). The male BALB/c nude mice (4–6 weeks) were randomly separated into two groups (*n* = 5 each group). Mia-PaCa2 cells encapsulated in the soft and stiff GelMA hydrogels were subcutaneously transplanted into the nude mice. Then the hydrogels were collected and sliced, and immunohistochemical staining of FA synthesis proteins (FASN, ACC, ACLY, SCD1) was carried out.

### Statistical analysis

Data were obtained from at least triplicates. *P*-values were acquired by two-tailed unpaired Student’s t-test analysis or one-way ANOVA analysis using GraphPad Prism 9. *P *< 0.05 was considered statistically significant (*P < 0.05, **P < 0.01 and ***P < 0.001), and data are represented the mean ± SD.

## Results and discussion

### Transcriptional landscape indicated that lipid metabolism was involved in the stiff group

The PDAC occurrence and progression are typically along with the alteration of energy metabolism, primarily glycolysis, amino acid and lipid metabolism [14]. Mounting evidence has suggested that the modification of energy metabolism exerted a noticeable impact on the malignant phenotype of carcinoma, including chemoresistance, stemness and metastasis. For instance, the lipid metabolic reprogramming of PDAC cells elicited the inhibition of invasive, migration and metastasis via diminishing lipid storage [15]. The tryptophan metabolism resulted in the enhancement of PDAC stemness and chemoresistance through BICC1-regulated 2,3-dioxygenase-1 [16]. Similarly, lipid and TG accumulation advanced gemcitabine resistance through post-transcriptional stabilization of TGFB2 [17].

**Figure 1.**
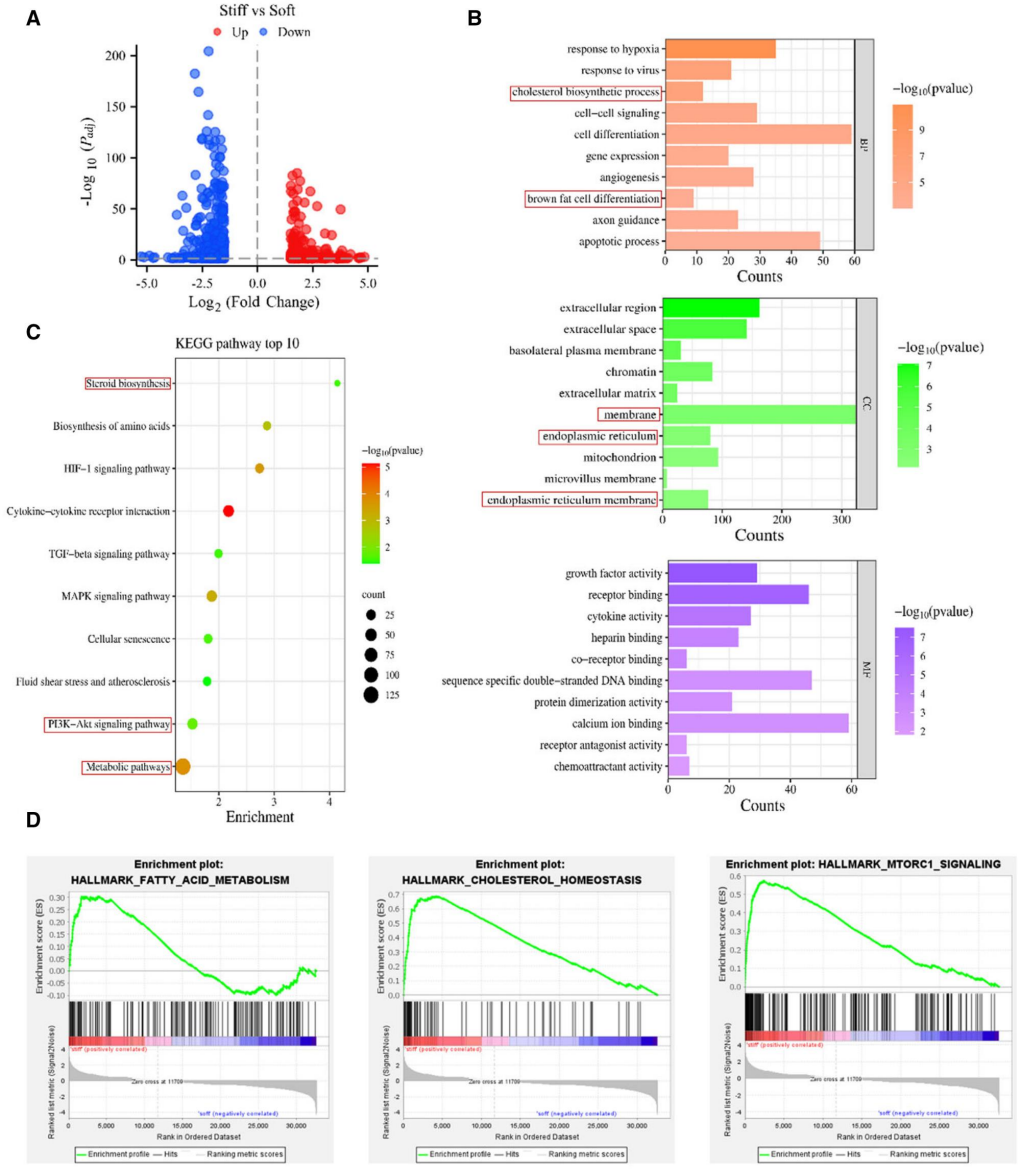
RNA-sequencing analysis of Mia-PaCa2 cells encapsulated in the soft and stiff groups indicated that matrix stiffness may regulate lipid metabolism. (**A**) Volcano plot representation of significant DEGs (*P*_adj_ < 0.05, |log_2_FC| > 1.5). Red: significantly highly expressed genes. Blue: genes with significantly low expression. (**B**) The top 10 GO enrichment analyses were displayed in the histogram plot. (**C**) The top 10 enriched KEGG pathways are showcased in the bubble plot. (**D**) GSEA identified “fatty acid metabolism”, “cholesterol homeostasis” and “mTORC1 signaling” upregulated in the stiff group.

To explore whether energy variation participates in the matrix stiffness-regulated PDAC chemoresistance, RNA sequencing was carried out with Mia-PaCa2 cells encapsulated in the soft and stiff groups. Differential expression genes (DEGs, *P*_adj_ < 0.05, |Log_2_FC| > 1.5) analysis manifested that 289 up-regulated genes and 344 down-regulated genes in the stiff group versus soft group (Figure 1A). Further analysis of DEGs with Gene Ontology (GO) and Kyoto Encyclopedia of Genes and Genomes (KEGG) was performed to provide underlying insights into the biological roles and biochemical pathways. GO enrichment showcased that the main Biological Process included “cholesterol biosynthetic process” and “brown fat cell differentiation.” Cellular Component demonstrated that “membrane,” “endoplasmic reticulum” and “endoplasmic reticulum membrane” were remarkably enriched (Figure 1B). It has been reported that lipid metabolism mainly occurs on the endoplasmic reticulum membrane. Molecular Function displayed that “receptor binding” and “calcium ion binding” were different between the soft and stiff groups. Moreover, metabolic-related pathways were identified as the notable enrichment by KEGG analysis, among them, the PI3K-Akt signaling pathway possessed a significant impact on cell metabolism. In pancreatic neuroendocrine neoplasms, lipid metabolism promoted tumorigenic effects through the activation of PI3K/Akt/mTOR pathway [18]. Additionally, “Steroid biosynthesis” was also enriched (Figure 1C), which indicated that matrix stiffness may induce the change of lipid metabolism. GSEA manifested that “cholesterol homeostasis”, “FA metabolism” and “hallmark mTORC1 signaling” were remarkably upregulated in the stiff group (Figure 1D). As an essential energy sensor, mTORC1 has been commonly linked to mechanical processes. The mTORC1 activation converts the phosphorylation state of diverse downstream targets to harmonize the transition from a quiescent status to proliferation. The elevated anabolic processes, such as lipid synthesis, as well as suppressing catabolic processes, including proteasome-dependent proteolysis and autophagy, play essential roles during the procedure [19]. The complex lipid metabolism contains large and diverse lipids and lipid-derived products [20]. The untargeted metabolomics of Mia-PaCa2 cells was further performed to analyze metabolites. OPLS-DA was utilized for modeling showcased that the soft and stiff groups exhibited significant differences (Supplementary Figure S1A). A multicriteria evaluation system (VIP >1, FC >2 and *P *< 0.05) was used to explore differential metabolites between the two groups, which was clearly illustrated by the volcano map (Supplementary Figure S1B). The hierarchical clustering was further conducted to display the different expression of metabolites in the two groups (Supplementary Figure S1C). The representative molecules correlated with FA metabolism, including nervonic acid, acetic acid, butanedioic acid and carboxylic acid. Subsequently, the metabolites mapped onto various metabolic pathways showcased that “biosynthesis of unsaturated FA” was enriched, although without significant differences (Supplementary Figure S1D). Comprehensively, combined with the omics results (RNA-seq and untargeted metabolomics), we speculated that lipid metabolism was involved in the process of matrix stiffness-mediated PDAC chemoresistance.

### Stiff matrix elevated FA synthesis of PDAC cells

**Figure 2.**
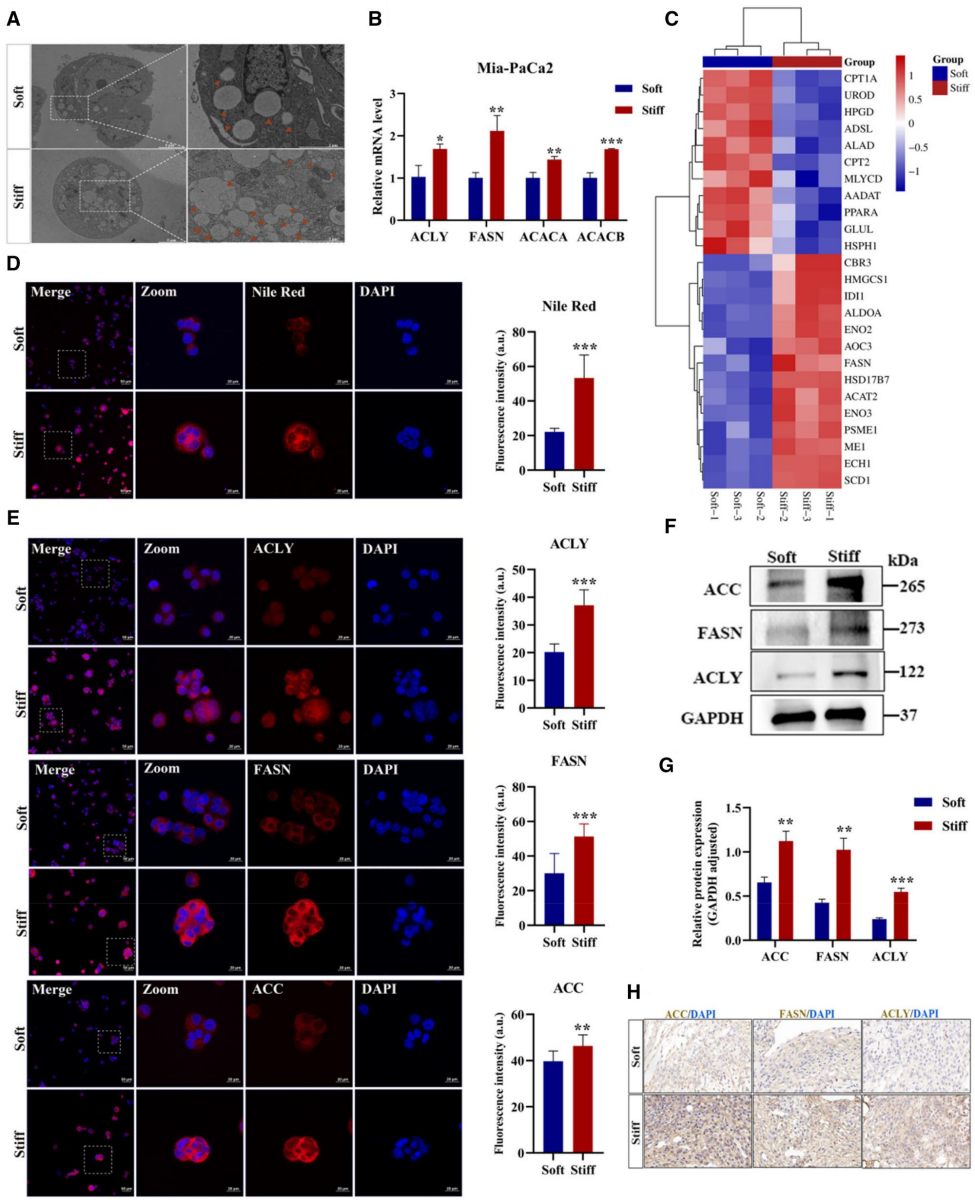
Matrix stiffness promotes neutral lipid accumulation through the upregulation of lipid synthetase expression in Mia-PaCa2 cells. (**A**) Representative TEM images, red arrows indicate LDs. Scale bar: 2 μm for high magnifications and 5 μm for low magnifications. Zoomed-in areas were shown in white squares. (**B**) The mRNA expression of FASN, ACLY, ACACA and ACACB. β-Actin as internal reference. (**C**) Heatmap of DEGs involved in fatty acid metabolism. (**D**) Cellular neutral lipids were determined by Nile Red and DAPI staining (red: LDs, blue: DAPI). (**E**) Representative immunofluorescent images of ACLY (top), FASN (middle) and ACC (bottom) (red: ACLY, FASN, ACC; blue: DAPI). Scale bar: 20 μm for high magnifications and 50 μm for low magnifications. Zoomed-in areas were shown in white squares. (**F, G**) The protein expressions and statistical analysis of ACLY, ACC and FASN. GAPDH as a reference protein. (**H**) Representative immunohistochemical staining images of ACC, ACLY and FASN on sections of Mia-PaCa2 cells encapsulated in the soft or stiff hydrogel after subcutaneous transplantation *in vivo*. Scale bar: 20 μm. Data were shown as the mean ± SD (*P < 0.05, **P < 0.01, ***P < 0.001).

The matrix stiffness of various tumors profoundly influences diverse malignant phenotypes, including proliferation, invasion, metastasis, angiogenesis, immune escape, as well as metabolic reprogramming [21, 22]. As a massive and diverse string of nutrients, lipids, comprised of lipoid and fat, are extensively distributed in cellular organelles [5]. Additionally, with sufficient energy supplies, lipids are stored in the intracellular LDs. As for nutrient scarcity, lipids function as energy sources and second messengers to trigger homeostasis via signal transduction mechanisms [23]. As energy-rich compounds, lipids play an essential role in the maintenance of cellular physiology by regulating lipid synthesis, uptake and degradation. During PDAC malignant progression, lipid metabolism contains FA oxidation, *de novo* lipogenesis and lipid profiles dynamics. It was well recognized as a potential therapeutic strategy for targeting lipid metabolism. The *de novo* synthesis provides the major source of FAs for tumor cells, rather than exogenous uptake, to promote tumorigenesis and development. Serving as vital cellular metabolic hubs, LDs were encased in the polar and amphipathic monolayer phospholipids, among them the core neutral lipids, primarily consisting of triacylglycerol and cholesterol esters. The abundant LDs in PDAC cells are conducive to sustain cell survival and proliferation under energy-stress conditions. The neutral lipids could be colored red with fat-soluble diazole dye Oil Red O, then the position and quantity of LDs were clearly displayed under a microscope. The staining results showcased that PDAC tissues exhibited more red region compared to the adjacent tissue (Supplementary Figure S2A). Owing to the ultra-high resolution, the TEM was further applied to observe the LDs with detailed parameters of the amount, size, shape and distribution. The results unveiled that the sum of LDs per cell was pronouncedly elevated in the stiff group of both Mia-PaCa2 and CFPAC-1 cells (Figure 2A and Supplementary Figure S3A). Acetyl-CoA is the unique carbon source and precursor for FAs biosynthesis in mammalian cells, which is generated from the precursor of citrate under the existence of ACLY [24]. ACC as a rate-limiting enzyme, including ACC1 and ACC2, exerted crucial role during the synthesis of numerous FAs [25]. Mainly expressed in lipogenic tissues, ACC1 is encoded by ACACA. ACC2 anchors to the outer mitochondrial membrane and encodes by ACACB, which governs the uptake of FAs and β-oxidation [26]. During the *de novo* production of FAs, ACC carboxylates acetyl-CoA to generate malonyl-CoA [27], which serves as a direct FA synthesis substrate and key regulator of FA catabolism. FASN condenses acetyl-CoA and malonyl-CoA molecules into palmitic acid, which favors the elaborate FA synthesis, the structure of the plasma membrane and the palmitoylation of protein [28]. Aim to elaborate on the influence of matrix stiffness on fatty acid synthesis, RNA levels of related genes were conducted. Our results indicated that ACLY, ACACA, ACACB and FASN expression were up-regulated in the stiff group of Mia-PaCa2 and CFPAC-1 cells (Figure 2B and Supplementary Figure S3B). The expression trend of FASN was coincident with the GSEA analysis result (Figure 2C). Nile Red binds to intracellular neutral lipids and emits red fluorescence. The staining results indicated that Mia-PaCa2 and CFPAC-1 cells of the stiff group possessed higher fluorescence intensity, similar to the result of Oil Red O staining (Figure 2D and Supplementary Figure S3C). Soft matrix strengthened lipid synthesis and storage during the adipogenic differentiation of stem cells through activating SREBP [29], which is inconsistent with our results, perhaps due to the different hydrogel system of fibronectin-coated polyacrylamide. Additionally, the average fluorescence intensity of ACLY, FASN and ACC exhibited distinct reinforcement in the stiff group versus soft group in both Mia-PaCa2 and CFPAC-1 cells (Figure 2E and Supplementary Figure S3D–F). Gray value statistical analysis results revealed distinctly higher expression of ACLY, FASN and ACC in the stiff group of Mia-PaCa2 cells, which was coincidence with the immunofluorescence results (Figure 2F and G). TG is generated via *de novo* FAs binding to a glycerol backbone under the catalysis of FASN [30]. Subsequently, the TG concentration of both cell lines was measured and further confirmed that the stiff group exhibited a higher level (Supplementary Figures S2B and S3G). For further validate whether the matrix stiffness promoted the FAs synthesis *in vivo*, both soft and stiff hydrogels embedded with Mia-PaCa2 cells were subcutaneous transplanted into the nude mice. The immunohistochemical staining results indicated that FASN, ACC and ACLY harbored higher protein expression in the stiff group, which agreed with the tendency of *in vitro* outcomes (Figure 2H). Taken together, stiff matrix augments lipid metabolism of PDAC through upregulating the lipogenic enzymes expression of ACLY, ACC and FASN.

### FA synthesis involved in 3D matrix stiffness-mediated PDAC chemoresistance

**Figure 3.**
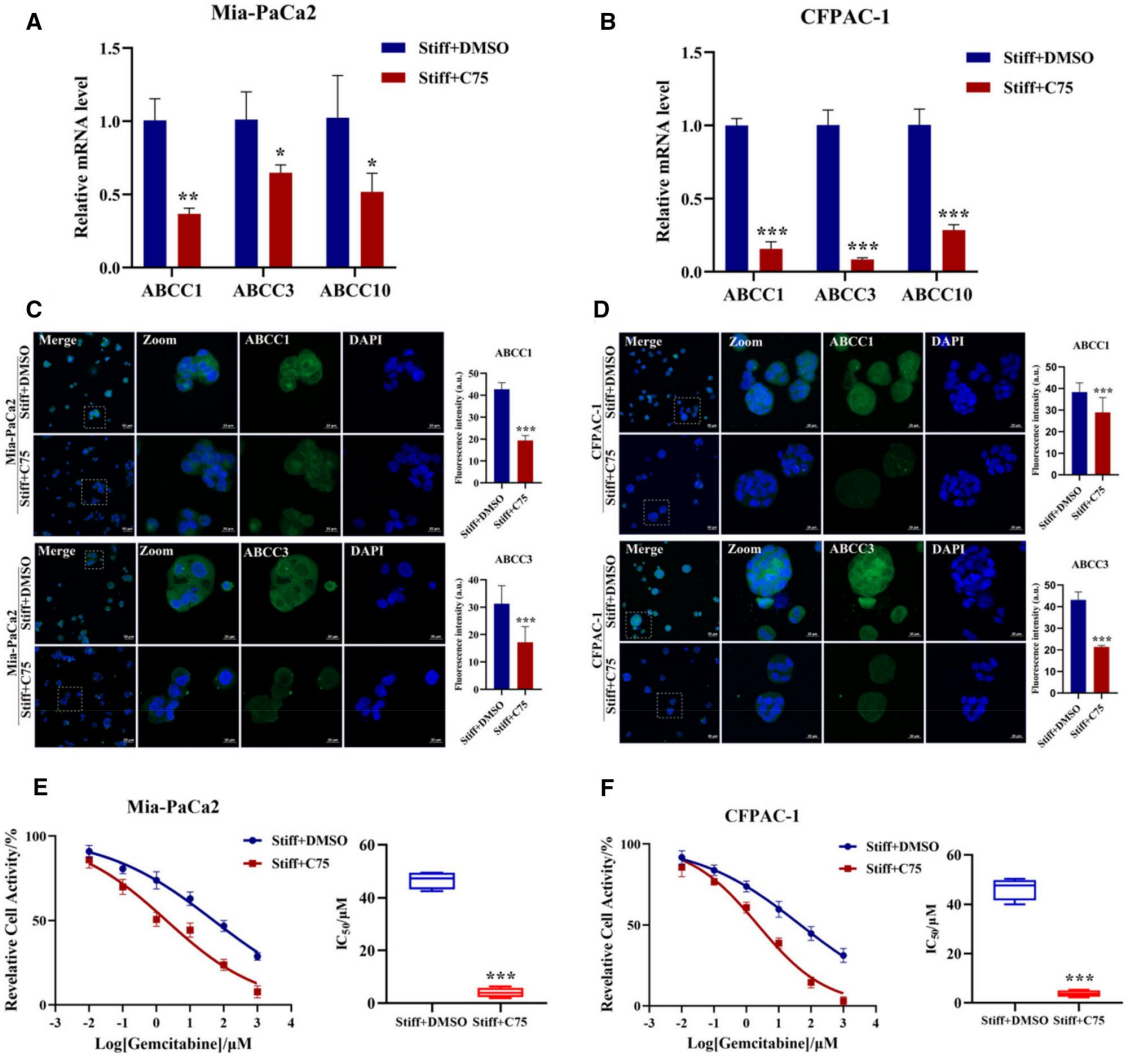
Matrix stiffness promotes gemcitabine resistance through mediating fatty acid synthesis of PDAC. Mia-PaCa2 and CFPAC-1 cells were both treated with FASN inhibitor C75 (5 μM, 48 h) or DMSO in the stiff group. (**A, B**) The mRNA expression of ABCC1, ABCC3 and ABCC10. β-actin as internal reference. (**C, D**) Representative immunofluorescent images of ABCC1 (top) and ABCC3 (bottom) (green: ABCC1, ABCC3; blue: DAPI). Scale bar: 20 μm for high magnifications and 50 μm for low magnifications. Zoomed-in areas were shown in white squares. (**E, F**) CCK-8 assay of gemcitabine sensitivity with increasing concentrations (0.01, 0.1, 1, 10, 100, 1000 μM, 5 replicates each) for 48 h (left). the statistical analysis of the IC_50_ value (right). Data were shown as the mean ± SD (*P < 0.05, **P < 0.01, ***P < 0.001).

The biophysical characteristics of the tumor microenvironment function significantly influence the success of PDAC chemoresistance, including the extracellular microenvironment (ECM) stiffness [31]. The stiffer ECM advances the evolution, invasion and chemoresistance of PDAC. Therefore, the influence of ECM stiffness on therapeutic response and underlying mechanisms is receiving increasing attention. The 2D matrix stiffness induces EMT and facilitates PDAC chemoresistance [32]. In hepatocellular carcinoma, the increasing matrix stiffness promotes proliferation and cisplatin resistance of cells cultured on polyacrylamide gels [33]. Our prior research based on 2D GelMA hydrogel confirmed that matrix stiffness promoted gemcitabine resistance in PDAC [2]. Most research remained limited to 2D culture ambience, and the underlying regulatory mechanism within 3D conditions is still scarce. The cells within a 3D setting could display more faithful morphology and molecular regulation close to natural states. Hence, to clarify the linkage between the matrix stiffness and chemoresistance in 3D conditions would be more intriguing. Previous studies have found that the 3D matrix stiffness-induced chemoresistance occurs as a result of the elevated expression of drug efflux transporters regulated by the interaction of CD44 receptor with hyaluronan [1]. A stiffening 3D self-assembling PA-E3Y(h) hydrogels enhance PDAC chemoresistance via inducing ECM deposition, promoting EMT and enriching the CD133^+^/CXCR4^+^ cells [34]. Blocking the lipid supply may have a potential impact on cancer biology behaviors. Emerging evidence positions metabolic reprogramming as a key mediator of stiffness-driven malignant phenotype in various tumors. For example, the exchange of aspartate and glutamate triggered squamous cell carcinoma progression and metastasis in a matrix stiffness-dependent manner [35]. In addition, matrix stiffening reprograms glutamine metabolism to elicit invasion in breast cancer through promoting the glutamylation and stabilization of microtubules [36]. Matrix stiffening also activates the MAPK-YAP mechano-transduction pathway to enhance aerobic glycolysis in HCC cells [37]. Besides, stiffer matrix promotes the activation of CLIC1 to bolster the Warburg effect via ROS/HIF-1α axis to drive PDAC proliferation [38]. Although glycolysis produces energy for the rapid proliferation of cancer cells for rapid proliferation [39], more energy and raw materials can be provided by lipid metabolism during the process of oncogenesis, development and metastasis [5]. Nevertheless, whether matrix stiffness regulates PDAC lipid metabolism to mediate chemoresistance is still blurred. Some studies have confirmed that PDAC chemoresistance can be regulated by lipid metabolism. For instance, abnormal lipid metabolism affects chemotherapy sensitivity; lipid raft cholesterol accumulation resulted in gemcitabine-resistance of PDAC by stabilizing SREBP-1c mRNA via coordination with HuR [40]. Decreased low-density lipoprotein level enables more sensitivity to PDAC chemoresistance [41]. Consequently, the role of FA synthesis in matrix stiffness-mediated PDAC chemoresistance was further explored. As the key metabolic enzyme of *de novo* lipogenesis, FASN was considered as a promising target in for various cancers. C75 (FASN inhibitor) interacts with the domain of enoyl reductase, β-ketoacyl synthase, as well as thioesterase [42], in turn inhibited the synthesis of FAs. ATP-binding cassette (ABC) transporter superfamily emerged as pivotal regulators of drug elimination and efflux, which consists of seven (A–G) subfamilies, including ABCC1, ABCC3 and ABCC10 [43, 44]. ABCC1 and ABCB1 are involved in gemcitabine resistance of PDAC [45]. Simultaneously, the suppression of ABCC1, ABCC2, ABCC3 and ABCC4 expression reinforced gemcitabine sensitivity of PDAC [46]. The treatment with C75 (5 μM, 48 h) of Mia-PaCa2 cells in the stiff group diminished the expression of lipid synthesis-related proteins (Supplementary Figure S4A). While for TG content and mRNA levels of ABCC1, ABCC3 and ABCC10, both Mia-PaCa2 and CFPAC-1 cells displayed a downward tendency in the stiff group (Supplementary Figure S4B and  Figure 3A and B). The immunofluorescence of ABCC1 and ABCC3 manifested the identical trend, with 0.46-fold, 0.58-fold in Mia-PaCa2 cells and 0.76-fold, 0.5-fold in CFPAC-1 cells, respectively (Figure 3C and D). Gemcitabine is a nucleoside analogue typically applied for the clinical therapy of PDAC tumors. To assay PDAC gemcitabine sensitivity, the designated concentration gradient (0.01, 0.1, 1, 10, 100, 1000 μM) of gemcitabine was added. The result showed that the IC_50_ value prominently diminished in the stiff group treated with C75 (4.08 ± 1.82 μM for Mia-PaCa2 and 3.77 ± 1.33 μM for CFPAC-1) compared with the DMSO group (46.63 ± 3.38 μM for Mia-PaCa2 and 46.37 ± 4.57 μM for CFPAC-1) (Figure 3E and F). As anticipated, with the FA synthesis inhibited, the gemcitabine resistance of PDAC was depressed. For liver metastatic colorectal cancer, matrix stiffening promotes bevacizumab resistance through the crosstalk of lipid metabolism between hepatic stellate cells and colorectal cancer cells [47], which provides a promising therapeutic target to reverse drug resistance. Totally, the matrix stiffness promotes FA synthesis to confer PDAC chemoresistance.

### SCD1 is essential for matrix stiffness-triggered chemoresistance through lipid metabolic reprogramming

**Figure 4.**
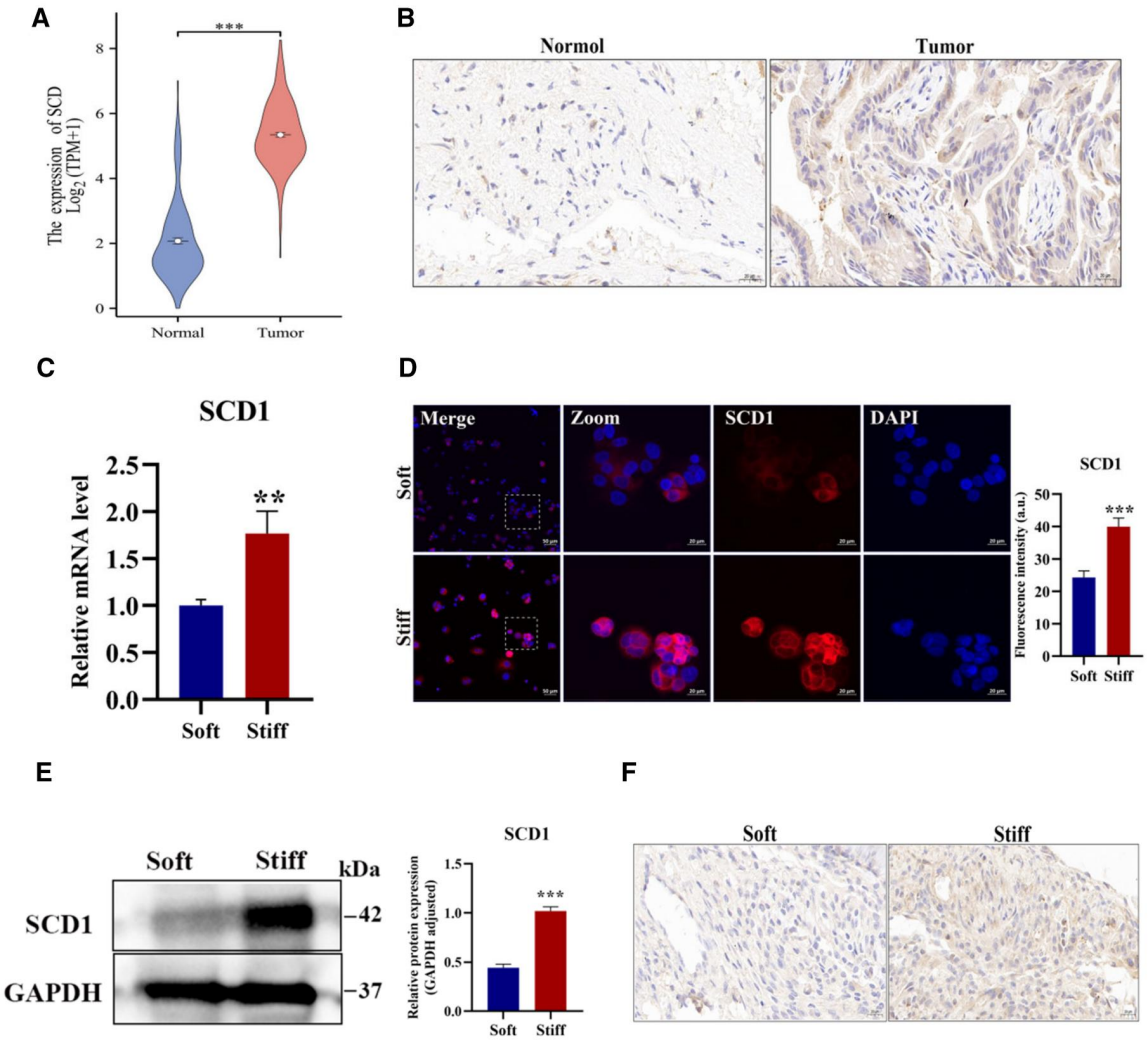
Matrix stiffness modulates SCD1 expression in Mia-PaCa2 cells. (**A**) Expression of SCD1 was markedly increased in tumor tissues (*n* = 179) compared with those in normal tissues (*n* = 171). (**B**) Representative immunohistochemistry staining images of SCD1 in tumor tissues and normal tissues. Scale bar: 20 μm. (**C**) The mRNA expression of SCD1. β-Actin as internal reference. (**D**) Representative SCD1 immunofluorescent images (red: SCD1; blue: DAPI). Scale bar: 20 μm for high magnifications and 50 μm for low magnifications. Zoomed-in areas were shown in white squares. (**E**) The protein expression and statistical analysis of SCD1. GAPDH as reference protein. (**F**) Representative SCD1 immunohistochemical staining images of Mia-PaCa2 cells encapsulated in the soft or stiff hydrogel after subcutaneous transplantation *in vivo*. Scale bar: 20 μm. Data were shown as the mean ± SD (*P < 0.05, **P < 0.01, ***P < 0.001).

The saturated fatty acids (SFAs) switch to monounsaturated fatty acids (MUFAs) rely on stearoyl-CoA desaturase (SCD), which commonly correlate with the cancer prognosis of patients [48]. As momentous substrates for cell membrane phospholipids, cholesterol esters and TG, MUFAs exerted major roles in the integrity and function of cellular structure. SCD1 is located in the ER and participates in a broad scope of neoplastic biological behaviors, for instance, proliferation, migration and metastasis [49], and is considered a promising target for therapeutics. The joint of SCD1 inhibition and low glucose distinctively impaired the metabolic plasticity of cancer cells [50]. The combined SCD1 inhibitor and ferroptosis inducer play a potential therapeutic role in ovarian cancer [51]. For bioinformation analysis, the clinical data were downloaded from TCGA (179 tumor tissues) and the GTEx database (171 normal tissues). The result revealed that the expression of SCD1 presented an obvious upregulation in tumor versus normal tissues (Figure 4A). The immunohistochemical staining of PDAC tissues showed that SCD1 was highly expressed with dark brown (Figure 4B). Aim to delve into the interplay between matrix stiffness and SCD1, the mRNA levels of SCD1 both in the soft and stiff groups were further carried out, which displayed distinctly elevated in the stiff group of Mia-PaCa2 and CFPAC-1 cells (Figure 4C and Supplementary Figure S5A). The stiff group presented 1.65-fold reinforced fluorescence intensity compared to the soft group for Mia-PaCa2 cells (Figure 4D), while for CFPAC-1 cells, with 2.31-fold (Supplementary Figure S5B). The western blot results of SCD1 also showcased significant augmentation in the stiff matrix of Mia-PaCa2 cells (Figure 4E). *In vivo* experiment further validated that Mia-PaCa2 cells embedded in the stiff hydrogel possessed higher SCD1 expression (Figure 4F), which was aligned with *in vitro* assay. Together, our results jointly suggested that a stiff matrix promoted the SCD1 expression.

**Figure 5.**
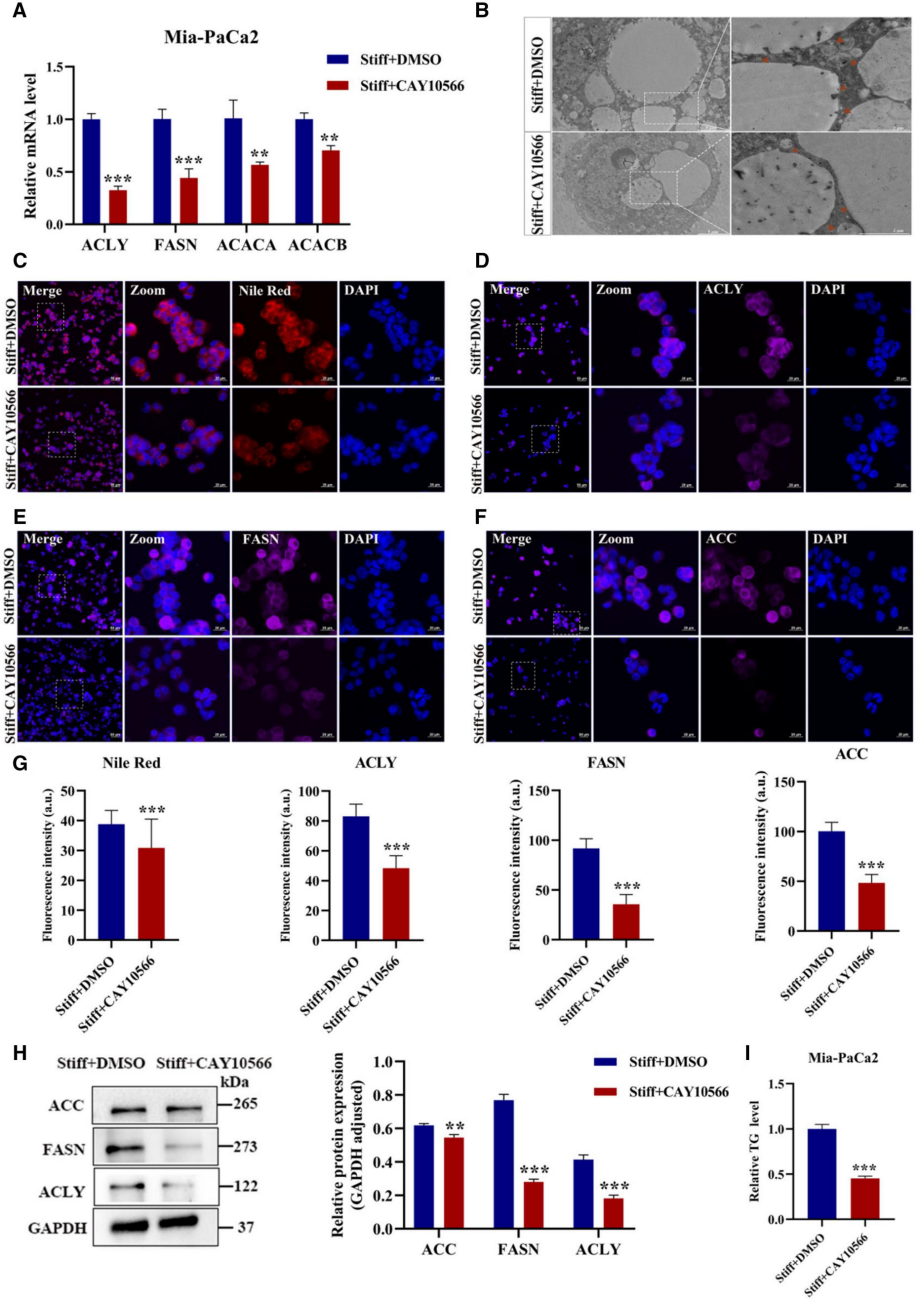
Matrix stiffness-induced fatty acid synthesis was suppressed in Mia-PaCa2 cells with pharmacological disruption of SCD1 activity. Cells were treated with DMSO or SCD1 inhibitor CAY10566 in the stiff group. (**A**) The mRNA expression of FASN, ACLY, ACACA and ACACB. β-Actin as internal reference. (**B**) Representative TEM images, red arrows indicate LDs. Scale bar: 2 μm for high magnifications and 5 μm for low magnifications. Zoomed-in areas were shown in white squares. (**C**) Cellular neutral lipids were determined by Nile Red and DAPI staining (red: LDs, blue: DAPI). (**D–F**) Representative immunofluorescent images of ACLY, FASN and ACC (purple: ACLY, FASN, ACC; blue: DAPI). Scale bar: 20 μm for high magnifications and 50 μm for low magnifications. Zoomed-in areas were shown in white squares. (**G**) Fluorescence quantitative analysis of Nile Red, ACLY, FASN and ACC. (**H**) The protein expressions and statistical analysis of ACLY, ACC and FASN. GAPDH as a reference protein. (**I**) Cellular TG content. Data were shown as the mean ± SD (*P < 0.05, **P < 0.01, ***P < 0.001).

**Figure 6.**
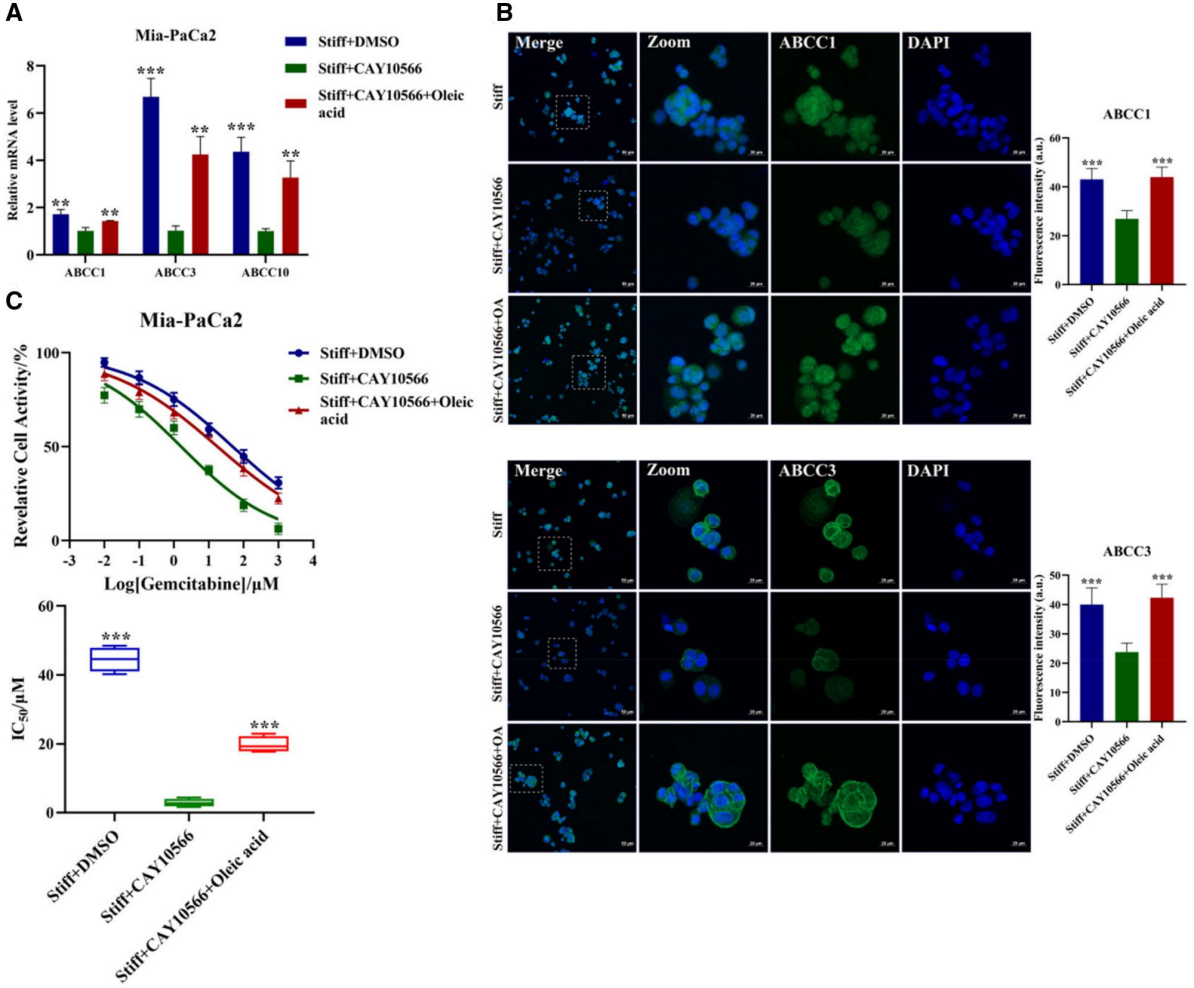
The addition of OA restored the chemoresistance of SCD1 pharmacological disruption in Mia-PaCa2 cells. Cells were treated with DMSO, SCD1 inhibitor CAY10566 only or CAY10566 and OA in the stiff group. (**A**) The mRNA expression of ABCC1, ABCC3 and ABCC10. β-Actin as internal reference. (**B**) Representative immunofluorescent images of ABCC1 (top), and ABCC3 (bottom) (green: ABCC1, ABCC3; blue: DAPI). Scale bar: 20 μm for high magnifications and 50 μm for low magnifications. Zoomed-in areas were shown in white squares. (**C**) CCK-8 assay of gemcitabine sensitivity with increasing concentrations (0.01, 0.1, 1, 10, 100, 1000 μM, 5 replicates each) for 48 h (left). The statistical analysis of the IC_50_ value (right). Data were shown as the mean ± SD (*P < 0.05, **P < 0.01, ***P < 0.001).

Whether SCD1 is involved in matrix stiffness-mediated FA synthesis was further performed due to it switches stearoyl CoA and palmitoyl CoA to OA and palmitoleic acid [52]. The stiff group was treated with CAY10566 (SCD1 inhibitor, 10 μM, 48 h) to pharmacologically disrupt the activity of SCD1. The gene expression of ACLY, FASN, ACACA and ACACB was diminished both in Mia-PaCa2 and CFPAC-1 cells (Figure 5A and Supplementary Figure S6A). Moreover, the TEM results showcased that the amount and size of LDs were distinctly decreased compared to the DMSO group (Figure 5B and Supplementary Figure S6B). The Nile red staining and the immunofluorescence of ACLY, FASN and ACC showcased that the cellular lipid accumulation was suppressed with the inhibition of SCD1 (Figure 5C–G and Supplementary Figure S6C–F). The protein level of ACLY (0.41-fold), FASN (0.36-fold) and ACC (0.89-fold) was markedly decreased in Mia-PaCa2 cells (Figure 5H). The cellular TG contents were reduced, with 0.45-fold and 0.38-fold in Mia-PaCa2 and CFPAC-1 cells, respectively (Figure 5I and Supplementary Figure S6G). Furthermore, the knockdown of SCD1 significantly attenuated FA synthesis in the stiff group of Mia-PaCa2 cells, with the diminishment of lipid synthesis-related protein expression and cellular lipid accumulation (Supplementary Figure S7A–D). These data indicated a vital role of SCD1 in mediating matrix stiffness-driven PDAC lipid metabolic reprogramming. Cancer cells with highly metastatic ability consumed OA to keep malignant phenotype, depending on the AMPK signaling pathway. Herein, the impact of OA on the matrix stiffness-induced chemoresistance was evaluated. Mia-PaCa2 cells in the stiff group were treated together with CAY10566 and OA, and the expression of ABCC1 and ABCC3 was reversed (Figure 6A and B). Meanwhile, the IC_50_ value of gemcitabine was also rescued, with elevated chemoresistance from 2.84 ± 1.14 μM to 19.77 ± 2.32 μM (Figure 6C). Notably, similar results were also obtained with SCD1 knockdown assay (Supplementary Figure S7E and F). The supplement of OA diminished the cytotoxic effect of osimertinib and gefitinib in non-small cell lung cancer [53]. Collectively, our results indicated that SCD1 participated in matrix stiffness-regulated FA synthesis and chemoresistance.

### The Piezo1 participated in matrix stiffness-mediated SCD1 expression through PI3K-Akt pathway

**Figure 7.**
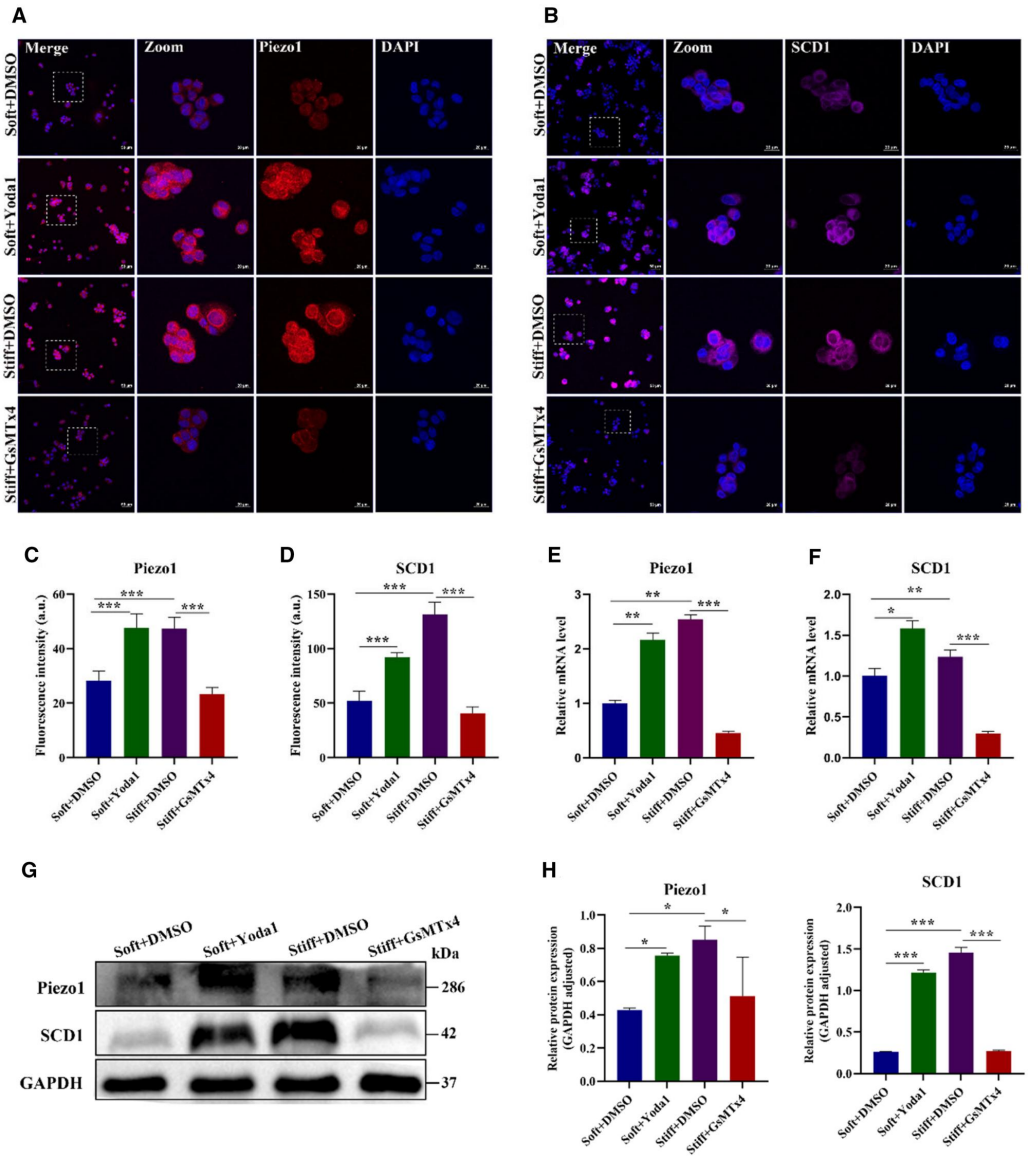
Matrix stiffness regulates SCD1 activation through Piezo1 in Mia-PaCa2 cells. Cells were treated with DMSO and Piezo1 agonist Yoda1 in the soft group or treated with DMSO and Piezo1 inhibitor GsMTx4 in the stiff group. (**A, B**) Representative immunofluorescent images of Piezo1 and SCD1 (red: Piezo1; purple: SCD1; blue: DAPI). Scale bar: 20 μm for high magnifications and 50 μm for low magnifications. Zoomed-in areas were shown in white squares. (**C, D**) Fluorescence quantitative analysis of Piezo1 and SCD1. (**E, F**) The mRNA expression of Piezo1 and SCD1. β-Actin as internal reference. (**G, H**) The protein expressions and statistical analysis of Piezo1 and SCD1. GAPDH as a reference protein. Data were shown as the mean ± SD (*P < 0.05, **P < 0.01, ***P < 0.001).

The signature of the extracellular network structure provides mechanical support during cell growth and tissue development. The resident cells successively perceive and respond to the intricate ECM milieu, including biochemical components and biophysical cues, to determine their fate through the interplay with ECM [54]. Cells perceive mechanical clues, such as stiffness, viscoelasticity, surface pattern, and dimensionality through membrane-bound receptors [55]. The complicated string of extracellular stimuli triggers intracellular signaling cascades and eventually transforms them into cellular responses. Mechanical stiffness mediates the alteration of metabolic activity through several key mechanosensitive molecules, including integrins and YAP [56–58]. FAK and integrin β1-pMLC-YAP signaling axis triggered by stiff matrix play a major role in platinum resistance [59]. Additionally, the soft matrix triggers integrin-linked kinase to induce chemoresistance through autophagy [60]. In breast cancer, matrix stiffness regulates drug resistance via YAP activation mediated by ILK [61]. Similarly, the YAP activation in triple-negative breast carcinoma cells is involved in ECM stiffness-mediated doxorubicin resistance [62]. However, the mechanism of mechanics-induced lipid metabolic reprogramming modulating PDAC chemoresistance is still less explored. Recently, the mechanosensitive ion channel protein Piezo1 has attracted increasing attention, which converts physical stimuli into electrochemical signals via accelerating Ca^2+^ influx and regulates various physiological processes [63]. Stiff matrix activated Piezo1 to increase the intracellular Ca^2+^ content, in turn modulated autophagy via the AMPK-ULK1 axis [64]. In gastric cancer, decreased Piezo1 enhanced the sensitivity to cisplatin and 5-fluorouracil [65]. The activation of Piezo1 may also be involved in the alteration of cellular metabolism. It has been reported that Piezo1 reinforced the transglutaminases activity of smooth muscle cells [66], and reinforced glycolysis of macrophages [67]. Glioblastoma multiforme cells with higher Piezo1 expression were resistant to temozolomide and more sensitive to TNF-related apoptosis-inducing ligand [68]. Therefore, we proposed that the activation of Piezo1 may participate in matrix stiffness-mediated lipid metabolic reprogramming and chemoresistance. Subsequently, the mRNA level and immunofluorescence assays were conducted to verify that Piezo1 is involved in matrix stiffness-mediated SCD1. As shown in Figure 7A and C, the fluorescence intensity of Piezo1 was 1.68-fold higher in the stiff group, and the gene expression level of Piezo1 also showcased a similar trend, with 2.54-fold (Figure 7E). The Ca^2+^ influx analysis of Mia-PaCa2 cells displayed that the fluorescence intensity of Ca^2+^ increased significantly in the stiff group (Supplementary Figure S8A). Piezo1 channel remains open by diminishing the activation threshold through Yoda1, a specific chemical agonist [69, 70]. GsMTx4 can inhibit a range of mechanosensitive ion channels, including Piezo1 [71]. Yoda1 and GsMTx4 were applied to further explore the function of Piezo1. GsMTx4 significantly reduced Piezo1 expression in the stiff group; its counterpart, Yoda1, remarkably promoted Piezo1 expression in the soft group. The treatment with Yoda1 increased Ca^2+^ influx, opposite trend with GsMTx4, which suggested that Ca^2+^ influx positively correlated with Piezo1 expression and matrix stiffness (Supplementary Figure S8B and C). In calcific aortic valve disease, Yoda1 pronounced increased Ca^2+^ concentration of the cytosolic, whereas Ca^2+^ influx was reduced with Piezo1 knockdown [72]. For Mia-PaCa2 cells, the fluorescence intensity of SCD1 was evidently diminished in the stiff group treated with GsMTx4, and pronounced augmented in the soft group intervened with Yoda1 (Figure 7B and D). The SCD1 mRNA expression was lower in the stiff group treated with GsMTx4 and higher in the soft group intervened with Yoda1 (Figure 7F). The protein expression levels of Piezo1 and SCD1 also showed a similar trend (Figure 7G and H). Overall, a stiff substrate stimulated the activation of Piezo1, promoting the expression of SCD1 through Ca^2+^ influx. It has been reported that the activation of the PI3K/Akt-SREBP1-SCD1 pathway augments the levels of MUFA-PLs and MUFA-ePLs [73]. The downward of Piezo1 substantially repressed the Ca^2+^ signal, in turn inhibiting the p-Akt [74]. Piezo1-related ILC3 activation partial attributed to the PI3K-Akt pathway in inflammatory bowel disease [75]. Due to the PI3K-Akt pathway was also enriched in our KEGG analysis, we speculated that matrix stiffness boosts SCD1 may via Piezo1 mediated the PI3K-Akt pathway. In order to determine the function of the PI3K-Akt pathway in Piezo1-regulated SCD1 activation, we treated Mia-PaCa2 cells with Yoda1 (Piezo1 promoter) in the soft group and GsMTx4 (Piezo1 inhibitor) in the stiff group. The results displayed that both Yoda1-induced and GsMTx4-suppressed influenced the expression of p-PI3K-Akt (Supplementary Figure S8D), which indicated that Piezo1 regulated the PI3K-Akt pathway. Then, the treatment with GsMTx4 or combined with GsMTx4 and SC79 (Akt promoter) in the stiff group showed that high expression of Akt reversed the low expression of SCD1 (Supplementary Figure S8E). In general, Piezo1 regulates SCD1 through the PI3K-Akt pathway.

## Conclusion

**Figure 8.**
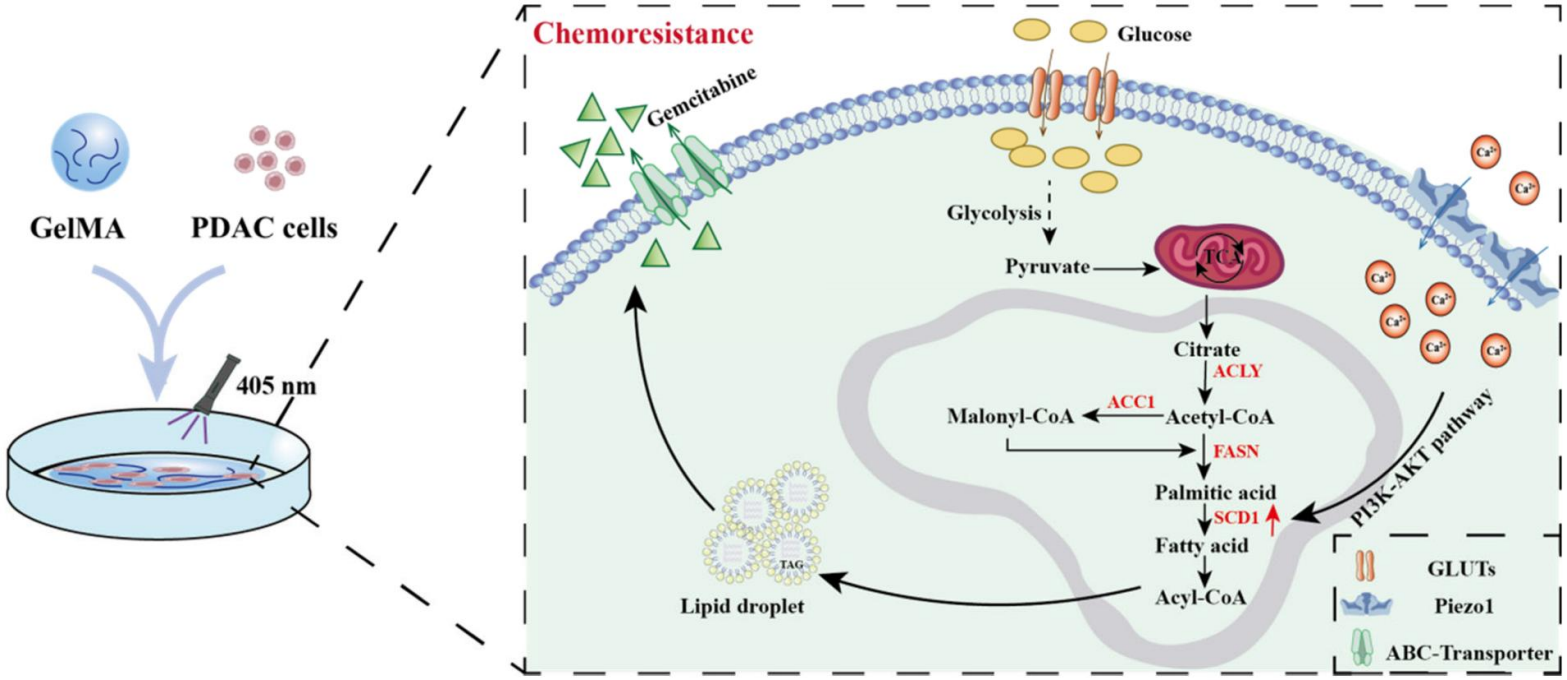
Schematic overview showing that 3D matrix stiffness elicited lipid metabolism reprogramming to regulate PDAC chemoresistance via the mechanosensitive ion channel Piezo1-mediated SCD1, and the PI3K-Akt pathway participated in Piezo1-mediated SCD1 expression.

Aim to illuminate the underlying mechanism of matrix stiffness-mediated PDAC chemoresistance, based on the dissection of the main component and stiffness of healthy and PDAC tissues, a 3D GelMA hydrogel with adjustable matrix stiffness was constructed. The transcription landscape and untargeted metabolomics analysis indicated the potential linkage between lipid metabolic reprogramming and matrix stiffness. Furthermore, matrix stiffness-regulated FA synthesis was validated through related enzyme expression, the content of LDs and cellular TG. Additionally, rescue assays elucidated that the PI3K-Akt pathway participated in Piezo1-mediated SCD1 upward. Taken together, our research deciphered that 3D matrix stiffness elicited lipid metabolic reprogramming to regulate PDAC chemoresistance via the Piezo1-mediated SCD1 (as illustrated in Figure 8). This study provides a supplement mechanism of PDAC chemoresistance from the perspective of matrix stiffness-mediated lipid metabolic reprogramming.

## Supplementary Material

rbaf056_Supplementary_Data
