# Supplementary material for: Matrix stiffness boosts PDAC chemoresistance via SCD1-dependent lipid metabolic reprogramming
Source: Regen Biomater. 2025 Jun 16;12:rbaf056. doi: 10.1093/rb/rbaf056 (PMC12308179; doi:10.1093/rb/rbaf056)
Supplement: rbaf056_Supplementary_Data [file rbaf056_supplementary_data.zip › Supporting information for publication.docx]

**Matrix stiffness boosts PDAC chemoresistance via**

**SCD1-dependent lipid metabolic reprogramming**

Xue Zhang^a, b #^, Biwen Zhu^a, c#^, Jiashuai Yan^a, c #^, Xi Chen^a, b^, Di Wu^a, c^, Zhen Wang^a, c^, Xiaoqi Guan^a, c^, Yan Huang^a, c^, Yahong Zhao^b^, Yumin Yang^b **^, Yibing Guo^a *^

^a^ Research Center of Clinical Medicine, Affiliated Hospital of Nantong University, Nantong University, Nantong 226001, P.R China.

^b^ Key Laboratory of Neuro-regeneration of Jiangsu and Ministry of Education, Co-innovation Center of Neuro-regeneration, Nantong University, Nantong 226001, P.R China.

^c^ Department of Hepatobiliary and Pancreatic Surgery, Affiliated Hospital of Nantong University, Medical School of Nantong University, Nantong 226001, P.R China.

Correspondence address: Tel: +86-513-85052612; Fax: +86-513-85052612; E-mail: guoyibing2008@163.com (Y.G.); Tel: +86-513-85511585; Fax: +86-513-85511585; E-mail: [yangym@ntu.edu.cn](mailto:yangym@ntu.edu.cn) (Y.Y.).

^#^ These authors contributed equally to this work.

**Supporting Information**.

**
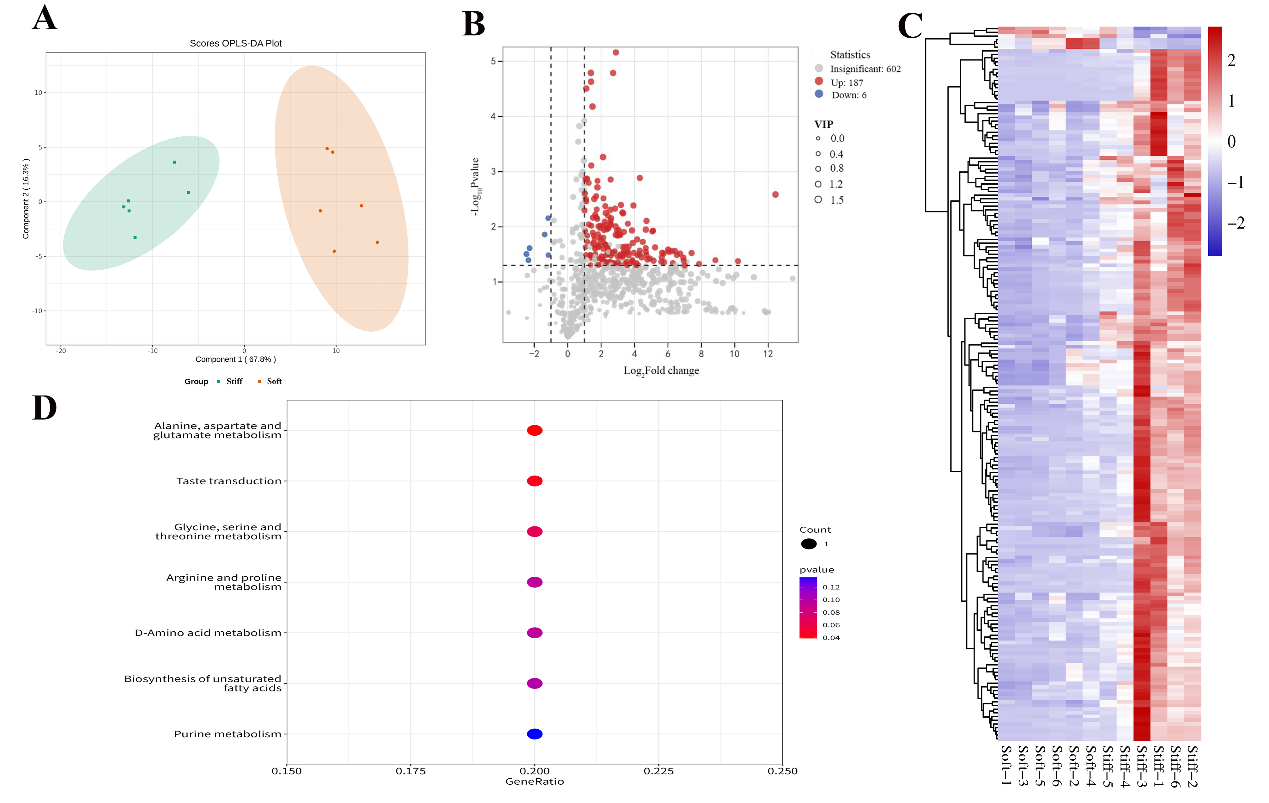
**

**Fig. S1 Metabolomics analysis of Mia-PaCa2 cells encapsulated in the soft and stiff groups.** (A) The OPLS-DA analysis chart. (B) The volcano plot of the differential expression metabolites. The red dots showed the significantly high expression, while blue dots mean distinctively low expression. Events in gray represented with non-significant differences. (C) The hierarchical clustering of different metabolites. The horizontal coordinate represented the group of samples, and the vertical coordinate represented the different metabolites. (D) Metabolic pathway enrichment map of differential metabolites.


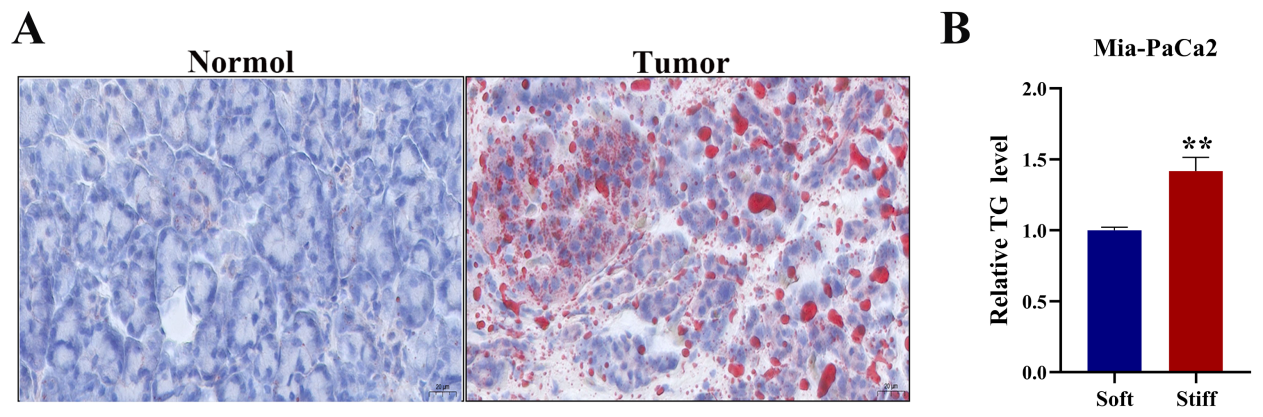


**Fig. S2 Tissue neutral lipids images and cellular triglycerides content.** (A) Representative images of Oil Red O staining in PDAC and adjacent tissues. Scale bars = 20 μm. (B) Cellular triglycerides content of Mia-PaCa2 cells in the soft and stiff groups. Data were shown as the mean ± SD (**p* < 0.05, ***p* < 0.01, ****p* < 0.001).


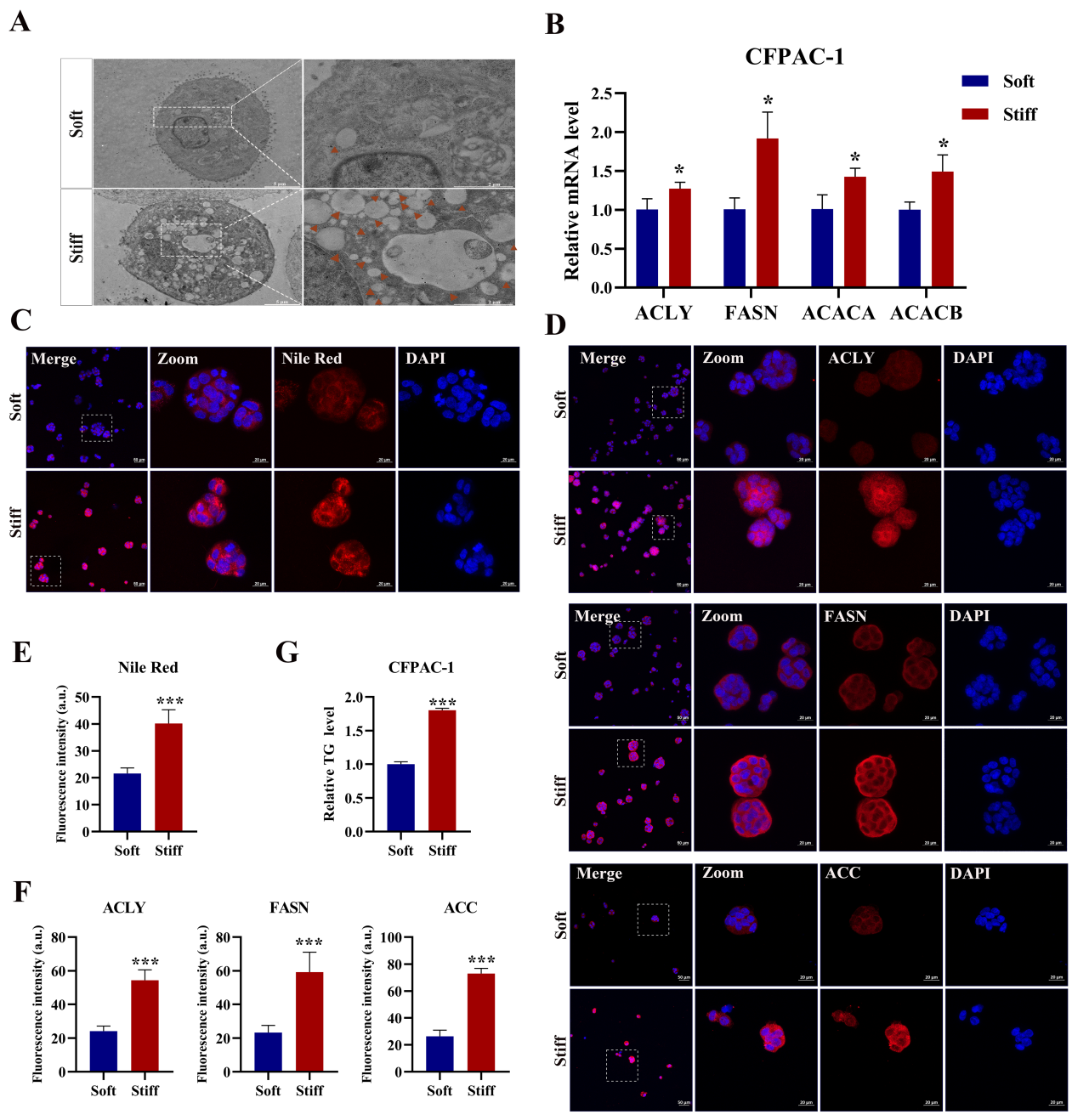


**Fig. S3 Matrix stiffness promotes fatty acid synthesis in CFPAC-1 cells**. (A) Representative TEM images, red arrows indicated lipid droplets. Scale bar: 2 μm for high magnifications and 5 μm for low magnifications. Zoomed-in areas were shown in white squares. (B) The mRNA expression of FASN, ACLY, ACACA, and ACACB. β-Actin as internal reference. (C) Cellular neutral lipids were determined by Nile Red and DAPI staining (red: lipid droplets, blue: DAPI). (D) Representative immunofluorescent images of ACLY (top), FASN (middle), and ACC (bottom) (red: ACLY, FASN, ACC; blue: DAPI). Scale bar: 20 μm for high magnifications and 50 μm for low magnifications. Zoomed-in areas were shown in white squares. (E-F) Fluorescence quantitative analysis of Nile Red, ACLY, FASN and ACC. (G) Cellular triglycerides content in the soft and stiff groups. Data were shown as the mean ± SD (**p* < 0.05, ***p* < 0.01, ****p* < 0.001).


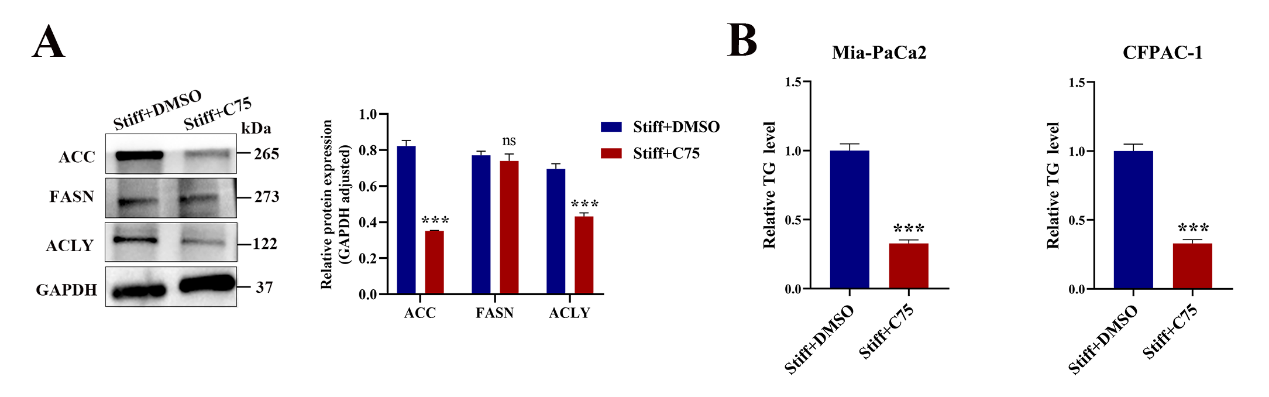


**Fig. S4 Fatty acid synthesis of PDAC cells treated with C75 in the stiff group**. (A) The protein expressions and statistical analysis of ACLY, ACC and FASN in Mia-PaCa2 cells. (B) Cellular triglycerides content of PDAC cells. Data were shown as the mean ± SD (**p* < 0.05, ***p* < 0.01, ****p* < 0.001).


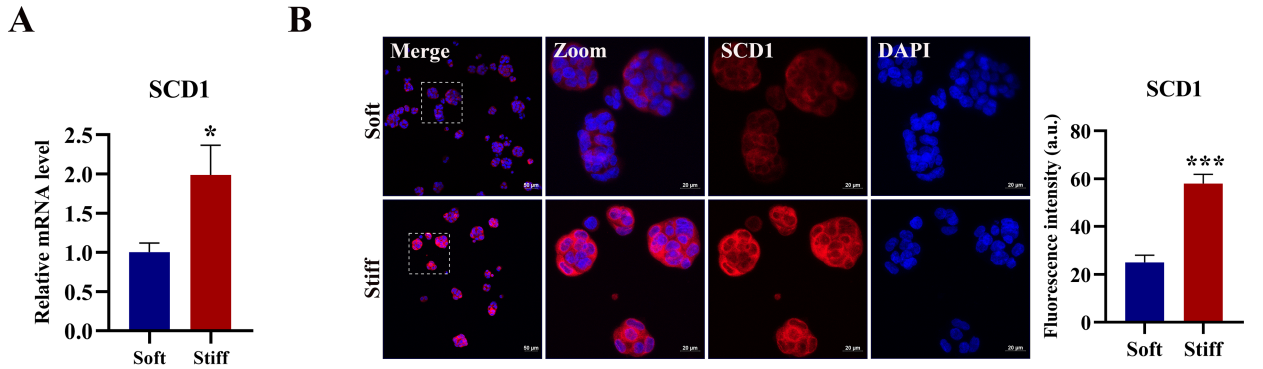


**Fig. S5** **Stiff matrix promoted the expression of SCD1 in CFPAC-1 cells.** (A) SCD1 mRNA expression in the soft and stiff groups. β-Actin as an internal reference. (B) Representative immunofluorescent images and quantitative analysis of SCD1 in the soft and stiff groups (red: SCD1; blue: DAPI). Scale bar: 20 μm for high magnifications and 50 μm for low magnifications. Zoomed-in areas were shown in white squares. Data were shown as the mean ± SD (**p* < 0.05, ***p* < 0.01, ****p* < 0.001).


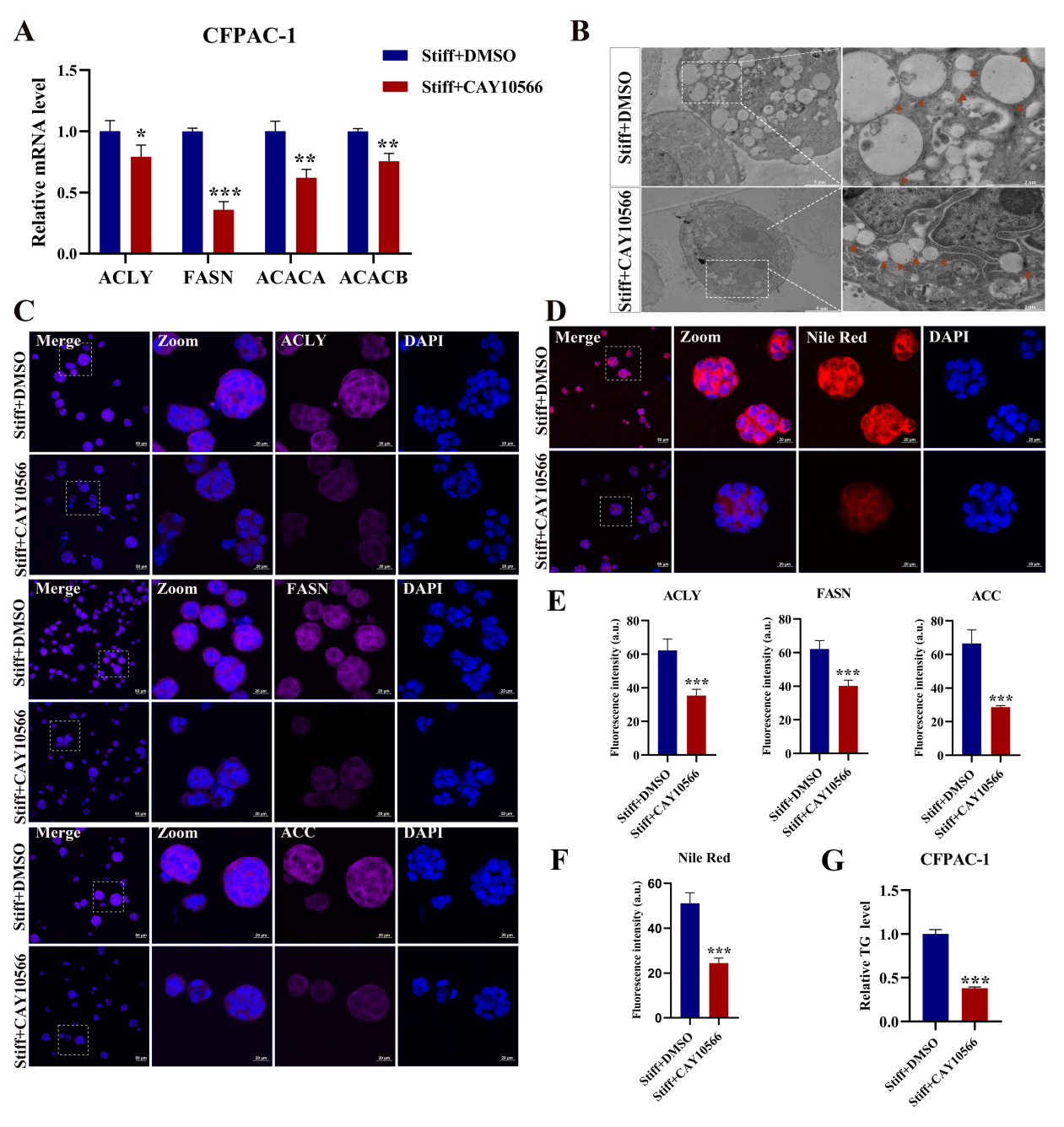


**Fig. S6** **SCD1 involved in matrix stiffness induced fatty acid synthesis in CFPAC-1 cells.** CFPAC-1 cells treated with DMSO or SCD1 inhibitor CAY10566 (10 μM, 48 h) in the stiff group. (A) The lipogenic enzyme mRNA expression of ACLY, FASN, ACACA, and ACACB. β-Actin as internal reference. (B) Representative TEM images, red arrows indicated lipid droplets. Scale bar: 2 μm for high magnifications and 5 μm for low magnifications. Zoomed-in areas were shown in white squares. (C) Representative immunofluorescent images of ACLY (top), FASN (middle), and ACC (bottom) (purple: ACLY, FASN, ACC; blue: DAPI). Scale bar: 20 μm for high magnifications and 50 μm for low magnifications. Zoomed-in areas were shown in white squares. (D) Cellular neutral lipids were determined by Nile Red and DAPI staining (red: lipid droplets, blue: DAPI). (E-F) Fluorescence quantitative analysis of Nile Red, ACLY, FASN and ACC. (G) Cellular triglycerides content. Data were shown as the mean ± SD (**p* < 0.05, ***p* < 0.01, ****p* < 0.001).


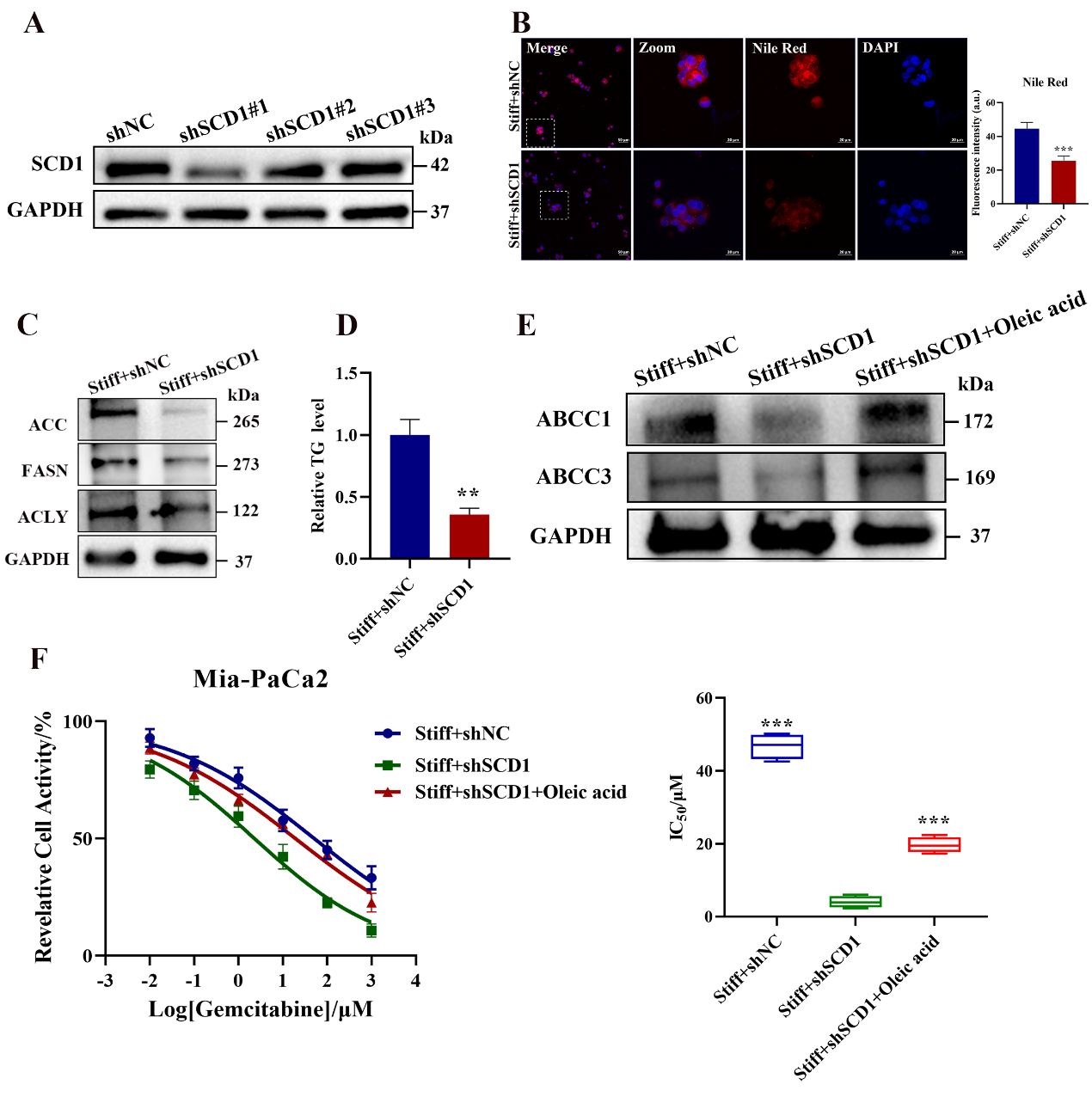


**Fig. S7 The SCD1 knockdown suppressed fatty acid synthesis, and SCD1 knockdown combined with oleic acid promoted chemoresistance in Mia-PaCa2 cells.** (A) Probed the SCD1 knockdown efficiency. (B) Cellular neutral lipids were determined by Nile Red and DAPI staining (red: lipid droplets, blue: DAPI). (C) The protein expressions of ACC, FASN and ACLY. (D) Cellular triglycerides content. (E) The protein expressions of ABCC1 and ABCC3. (F) CCK-8 assay of gemcitabine sensitivity with increasing concentrations (0.01, 0.1, 1, 10, 100, 1 000 μM, 5 replicates each) for 48 h (left). The statistical analysis of IC_50_ value (right). Data were shown as the mean ± SD (*p < 0.05, **p < 0.01, ***p < 0.001).


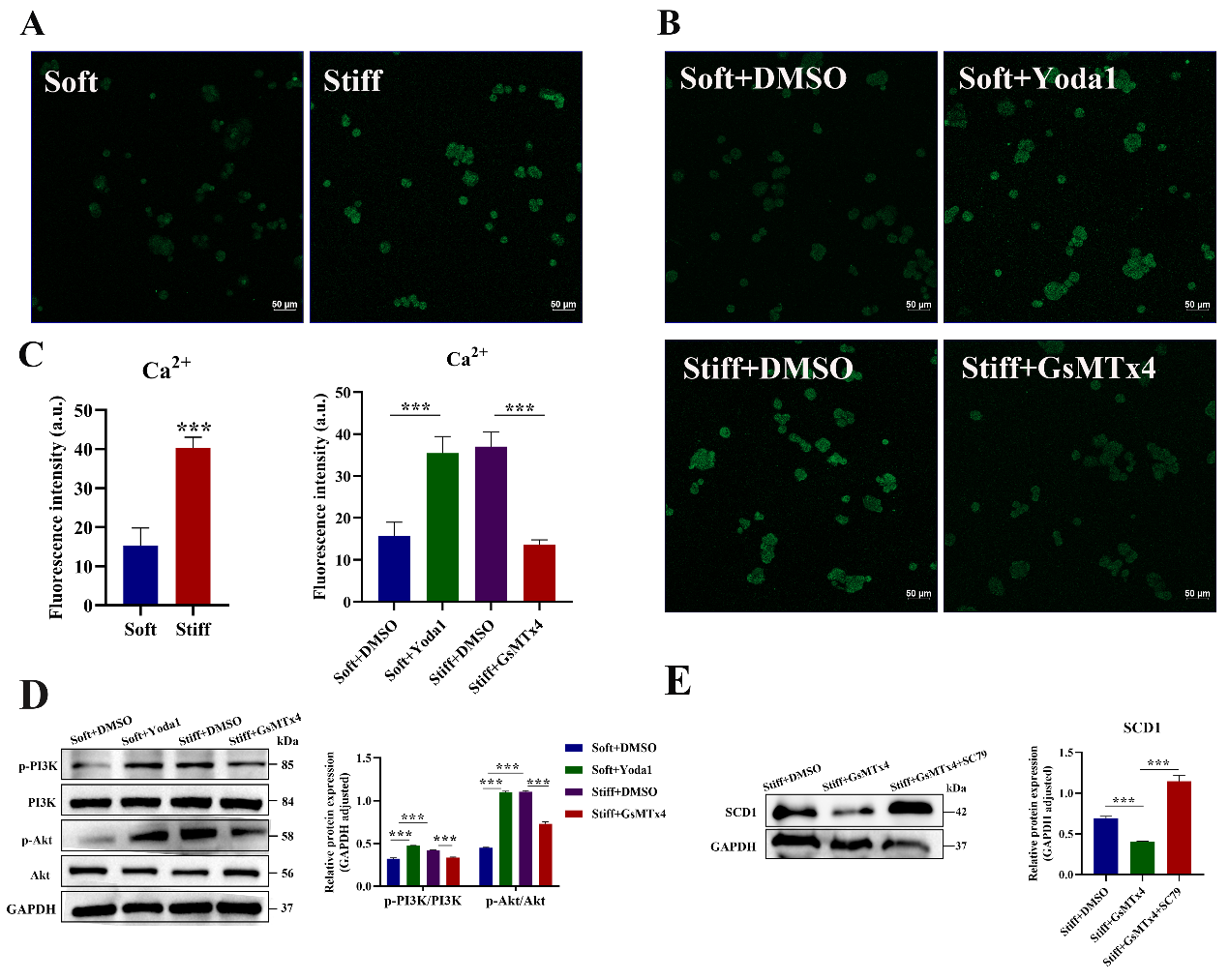


**Fig. S8 Matrix stiffness promoted the intracellular Ca^2+^ influx and activated PI3K-Akt pathway in Mia-PaCa2 cells.** Cells were treated with DMSO and Piezo1 agonist Yoda1 in the soft group, DMSO and Piezo1 inhibitor GsMTx4 or Piezo1 inhibitor GsMTx4 and SC79 in the stiff group. (A) Representative immunofluorescent images of Ca^2+^ in the soft and stiff groups. Scale bar: 50 mm. (B) Representative immunofluorescent images of cells treated with Yoda1, DMSO, or GsMTx4 in the soft or stiff groups. Scale bar: 50 mm. (C) Fluorescence quantitative analysis of Ca^2+^. (D-E) The protein expressions and statistical analysis of p-PI3K, PI3K, p-Akt, Akt and SCD1. Data were shown as the mean ± SD (**p* < 0.05, ***p* < 0.01, ****p* < 0.001).

**Table. S1**

| **Gene** | **Sequence (5’ - 3’)** | **Length** |
| --- | --- | --- |
| **β-Actin** | **F CGGAGGAACCACCATGTACC**  **R GCAGCCTTCACAGAGGCAAAT** | **20**  **21** |
| **FASN** | **F CCATCTACATCGACACCAG**  **R CTTCCACACTATGCTCAGGTAG** | **22**  **22** |
| **ACLY** | **F CAGAATCGGTTCAAGTATGCTC**  **R AAGTTTTCCACGACGTTTGATC** | **22**  **22** |
| **ACACA** | **F TGCCTCCACACTCACTCTTCC**  **R ACCTGCTGCCTGTCTACACTC** | **21**  **21** |
| **ACACB** | **F GGAGCACAGAGGCGGAGAG**  **R CAGGCGAGGAAGCAACAGTAAC** | **19**  **22** |
| **ABCC1** | **F ATCACCATCATCCCCCAGGA**  **R TGCAGTCCTCGAACTGTGTC** | **20**  **20** |
| **ABCC3** | **F TCAAGATGGTCCTGGGTGTCCTG**  **R GATGTAGAAGGTGGTGAAGCGGAAG** | **23**  **25** |
| **ABCC10** | **F TGGAGGAGGAGCAGAGCACATC**  **R CAATGGTCGCATACACGGTGAGG** | **22**  **23** |
| **Piezo1** | **F GCATCTTTCTCAGCCACTACTTC**  **R CCAGGGACTTCTCCTCAATCT** | **23**  **21** |
| **SCD1** | **F CTTTCTGATCATTGCCAACACA**  **R TGTTTCTGAAAACTTGTGGTGG** | **22**  **22** |

**Table. S1** Primer sequences of β-Actin, FASN, ACLY, ACACA, ACACB, ABCC1, ABCC3, ABCC10, Piezo1 and SCD1.
